# Integrated Virtual Screening Approach Identifies New CYP19A1 Inhibitors

**DOI:** 10.1021/acs.jcim.5c00204

**Published:** 2025-03-19

**Authors:** Sijie Liu, Jie Wu, Ya Chen, Clemens Alexander Wolf, Matthias Bureik, Johannes Kirchmair, Mario Andrea Marchisio, Gerhard Wolber

**Affiliations:** † Pharmaceutical and Medicinal Chemistry (Computer-Aided Drug Design), Institute of Pharmacy, Freie Universität Berlin, 14195 Berlin, Germany; ‡ Department of Pharmaceutical Sciences, Division of Pharmaceutical Chemistry, Faculty of Life Sciences, University of Vienna, Josef-Holaubek-Platz 2, 1090 Vienna, Austria; § School of Pharmaceutical Science and Technology, Tianjin University, Tianjin 300072, China

## Abstract

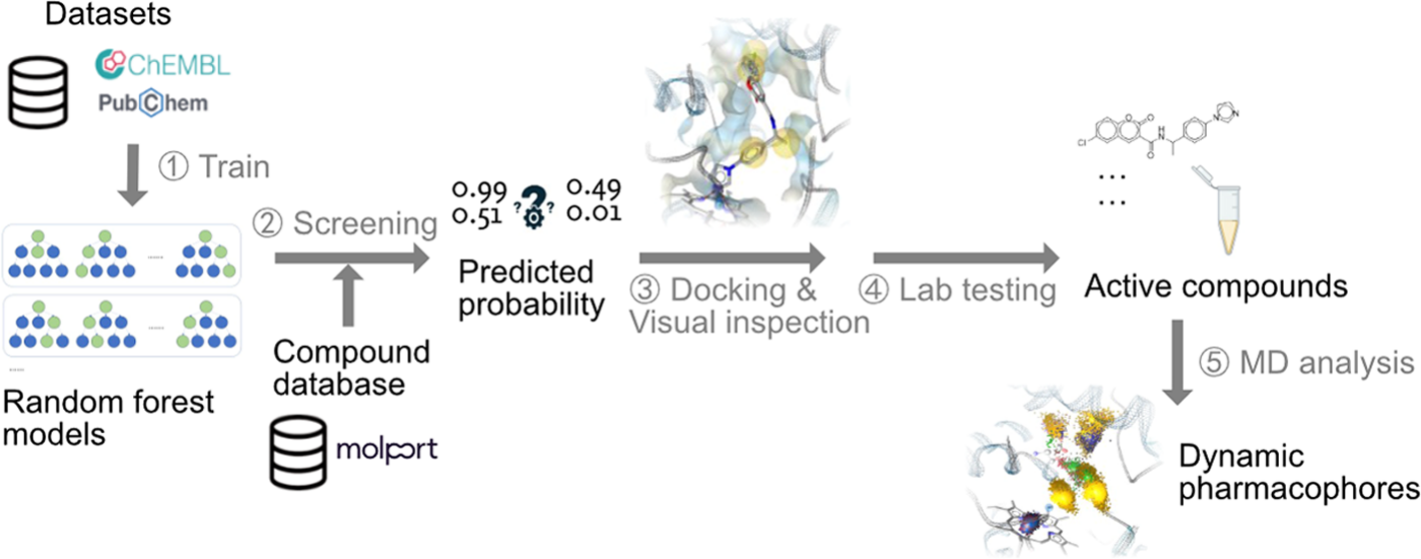

The human cytochrome P450 19A1 (CYP19A1, aromatase) is a heme-containing protein catalyzing the final steps of the biosynthesis of the steroid hormone 17β-estradiol. It is a key target for the treatment of sex-hormone-related disorders due to its role in mediating the conversion of androgens to estrogens. Here, we report the development of a virtual screening workflow incorporating machine learning and structure-based modeling that has led to the discovery of new CYP19A1 inhibitors. The machine learning models were built on comprehensive CYP19A1 data sets extracted from the ChEMBL and PubChem Bioassay databases and subjected to thorough validation routines. Ten promising hits that resulted from the virtual screening campaign were selected for experimental testing in an enzymatic assay based on heterologous expression of human CYP19A1 in yeast. Among the seven structurally diverse compounds identified as new CYP19A1 inhibitors, compound **9**, a novel, noncovalent inhibitor based on coumarin and imidazole substructures, stood out by its high potency, with an IC_50_ value of 271 ± 51 nM.

## Introduction

**Figure 1:**
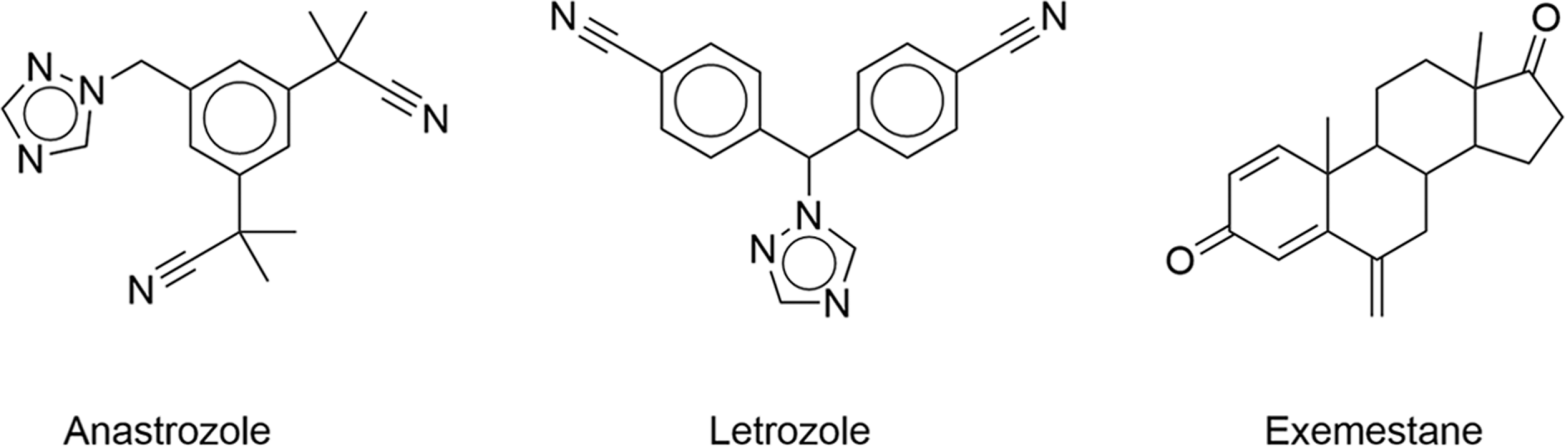
Commonly prescribed third-generation aromatase inhibitors.

Human aromatase, also known as human CYP19A1, is a member of the cytochrome P450s (CYPs), a superfamily of monooxygenases that contain heme as a prosthetic group and play essential roles in drug metabolism and homeostasis.^1^ Aromatase is responsible for the last step of the endogenous biosynthesis of estrogens in both men and women, as it catalyzes the aromatization of androgens (androstenedione/testosterone) to estrogens (estrone/17β-estradiol).^2^ Estrogens are a group of steroid hormones that, in addition to their physiological function in the development and regulation of the female reproductive system and secondary sex characteristics, also play a crucial role in the development and progression of breast cancer (the most common cancer among women worldwide) by promoting cell division and growth in breast tissue. Therefore, aromatase has been targeted for the treatment of ER+ (estrogen receptor positive) breast cancer, especially in postmenopausal women.^3^ In addition to breast cancer, other hormone-related conditions, such as short stature in boys,^4^ infertility in men,^5^ and women,^6^ endometriosis,^7^ leiomyomatosis,^8^ and Klinefelter’s syndrome^9^ are increasingly being treated by administering aromatase inhibitors. Several generations of aromatase inhibitors have been marketed and widely employed in clinical practice, with the third generation of aromatase inhibitors (Figure 1) being nowadays the most commonly used.

Aromatase inhibitors are commonly divided into two types: nonsteroidal aromatase inhibitors (NSAIs; type 1), such as anastrozole and letrozole, and steroidal aromatase inhibitors (SAIs; type 2), such as exemestane.^10^ While pharmacological therapies with AIs are useful adjuvants in treating the conditions mentioned above, there is increasing evidence that systemic suppression of aromatase can lead to adverse health effects, including musculoskeletal symptoms,^11^ cognitive dysfunction, and other neurological symptoms.^12^ In light of these challenges, it would be highly beneficial to identify new inhibitors with significantly lower permeation of the blood-brain barrier (BBB).^13^ In addition to the adverse effects that have been previously discussed, another problematic issue that arises during aromatase inhibitor administration is drug resistance. This issue can be divided into two categories: innate resistance and acquired drug resistance. The latter can occur as a result of continued treatment with aromatase inhibitors in breast cancer patients.^14^ Consequently, there is a need for novel aromatase inhibitors, designed to mitigate side effects or drug resistance and tailored to individual patient profiles. Furthermore, these inhibitors should demonstrate the potential for use in conjunction with various adjunctive therapies, aiming to improve tolerability and reduce the incidence of side effects compared to current treatment protocols.

Computer-aided drug design (CADD) strategies, specifically tailored to the discovery of CYP19A1 inhibitors, have been extensively utilized to streamline drug development. High-quality structures of CYP19A1 in complex with substrates or inhibitors have been resolved down to 2.75 Å resolution.^15−17^ These structures provide crucial insights into key binding residues and their roles in catalysis. Molecular docking techniques have proven invaluable in elucidating structure–activity relationships (SARs) for potential CYP19A1 inhibitors, as demonstrated in studies of sulfonamide derivatives, where docking was used to predict SARs as CYP19A1 inhibitors.^18^ Furthermore, docking has been utilized to expedite the identification of aromatase inhibitors, facilitating the ranking of compounds based on their predicted binding affinities and interactions with the active site.^19^ Additionally, molecular dynamics (MD) simulations have been employed not only for binding energy calculations^20^ but also to analyze the SAR and stability of CYP19A1 inhibitor-protein complexes, offering insights into how dynamic protein–ligand interactions influence inhibitor potency.^21^

In addition to structure-based approaches, machine learning models are particularly useful as ligand-based approaches, as they permit the prediction of aromatase inhibitors from a range of scaffolds, including nonsteroidal and steroidal inhibitors. Although some research has investigated the potential of machine learning in predicting CYP19A1 inhibitors,^22−24^ incorporating such models in structure-based virtual screening pipelines remains an underexplored area.

The development of synthetic biology has provided powerful tools for the expression and application of heterologous proteins.^25^ Heterologous proteins generally have the characteristics of low cost, good specificity, suitability for analysis in complex environments, and simple operation; based on these advantages, heterologous proteins have broad application prospects in clinical diagnosis, food and drug analysis, and research on biomaterial.^26^ The CYP19A1 inhibitors screening and evaluation method based on heterologous expression of human CYP19A1 in yeast was adopted in this study to validate candidate inhibitors predicted by our machine learning model.^27^ This method allows the screening of a large number of candidate chemicals faster and more economically than other methods.^28−30^

In our work, we trained machine learning models on data from the ChEMBL database^31^ and PubChem^32^ BioAssay to predict the probability of small molecules to inhibit CYP19A1. These models were applied for virtual screening in combination with additional filtering strategies, including docking and visual inspection. We developed classifiers optimized for early enrichment, making them more suitable for virtual screening than previous classification models for CYP19A1 inhibition. We utilized an enzymic assay based on heterologous protein to evaluate the activity of the proposed CYP19A1 inhibitors identified through *in silico* modeling in our study. The screening process yielded several active compounds with previously unknown scaffolds, among which the most promising hit was then subjected to further investigation through structure-based modeling and MD simulations to understand its binding interactions better.

## Results and Discussion

### Analysis of the Data Available for Model Development and Validation

To build predictive models for aromatase (CYP19A1) inhibition, reliable sources of CYP19A1 inhibition activity data are essential. The ChEMBL^33^ database is a curated resource that compiles bioactivity data from scientific literature and assays, making it a key source of activity data in drug discovery. PubChem^32^ BioAssay is a comprehensive repository of bioactivity results from high-throughput screening and other experimental studies. We compiled data on the inhibition of human CYP19A1 by drug-like small molecules from the ChEMBL database and the PubChem BioAssay database, adhering to the protocol described in the Methods section. For the data set extracted from the ChEMBL database, compounds with pChEMBL values ≥6 were labeled as active compounds, and compounds with pChEMBL values <6 were labeled inactive. For data extracted from the PubChem BioAssay database, compounds with a PUBCHEM_ACTIVITY_SCORE of 0 were labeled as inactive, compounds with scores between 40 and 100 were labeled as active, and compounds with a score outside this range were excluded from the study. The final data sets were composed of several hundred compounds, as summarized in Table 1.

**Table 1:** Datasets Used in This Study

	ChEMBL-derived data set^34^		PubChem BioAssay 743139-derived data set^35^	
	# active compounds (pChEMBL value ≥6)	# inactive compounds (pChEMBL value <6)	# active compounds	# inactive compounds
number of compounds	1361	1123	379	7562
number of compounds after preprocessing	756	609	196	2152
training set	610	482	163	1715
test set	146	127	33	437

**Figure 2:**
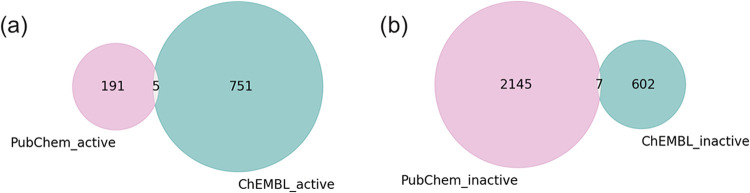
Venn diagrams illustrating the overlaps between the curated (a) active and (b) inactive data sets derived from the ChEMBL database (turquoise) and PubChem Bioassay (pink).

The Venn diagrams in Figure 2 show minimal overlap between the curated data sets derived from the ChEMBL database and PubChem BioAssay database. Only five aromatase inhibitors and seven inactive compounds are shared between the two data sets. The small overlap underscores the substantial differences between the data sets, which could contribute to distinct model preferences and performance when trained on each data set.

**Figure 3:**
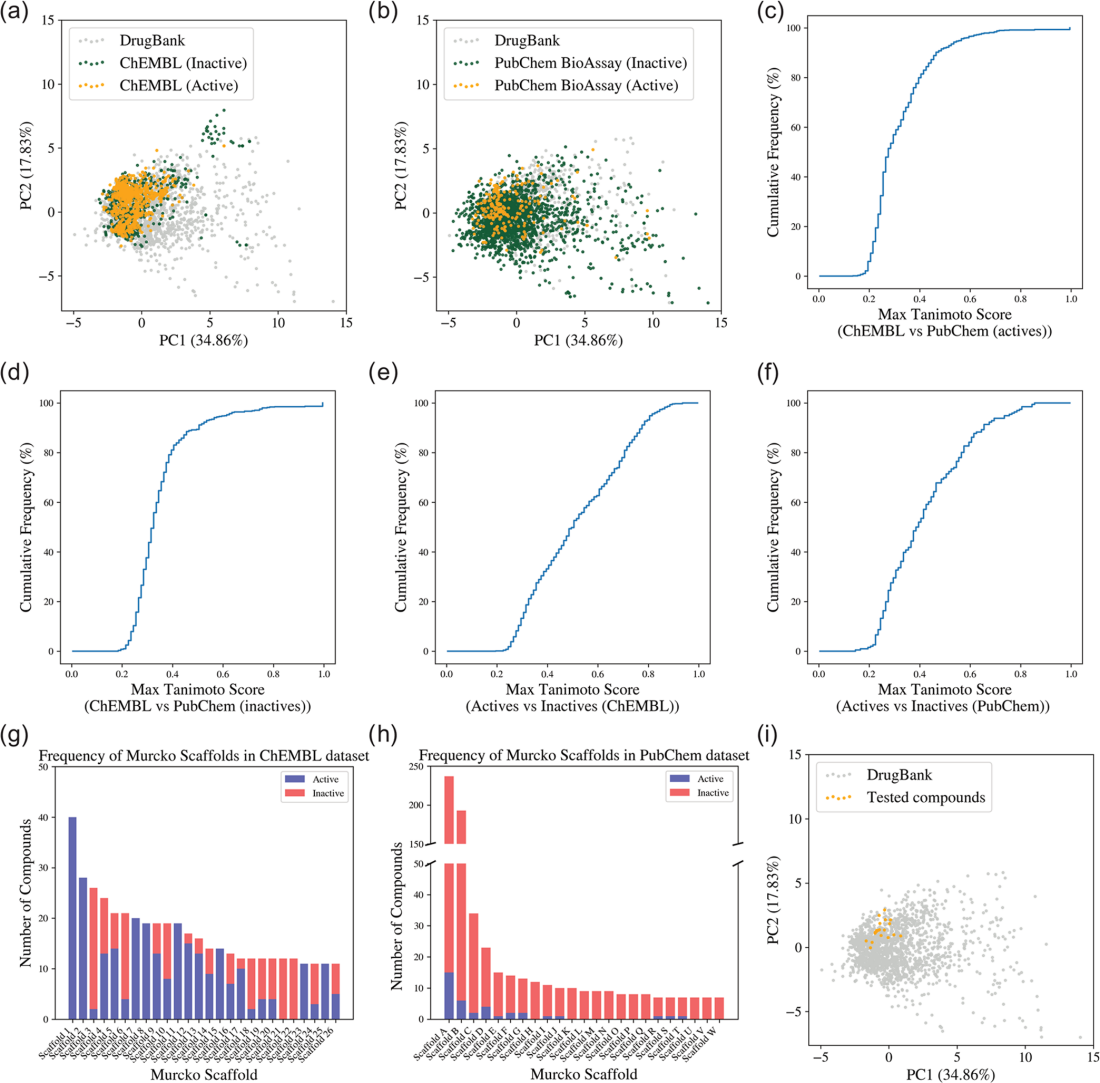
Chemical space analysis of the ChEMBL and PubChem BioAssay-derived data sets. (a, b) PCA scatter plots comparing the chemical space of active and inactive compounds in the (a) ChEMBL derived data set and (b) PubChem BioAssay data set with approved drugs from the DrugBank data set as a background. (c–f) Proportion of active or inactive compounds in the ChEMBL and PubChem BioAssay data sets at a given minimum similarity of nearest neighbors in the other group, based on the Tanimoto coefficient calculated from ECFP4 (2048 bits). (g) Number of molecules in the ChEMBL data set represented by the most prominent Murcko scaffolds. (h) Number of molecules in the PubChem data set represented by the most prominent Murcko scaffolds. The structures and further information for the scaffolds listed in (g, h) are listed in Table S1. (i) PCA scatter plot comparing the chemical space of the experimentally tested compounds in this study with approved drugs from the DrugBank data set as a background.

To better understand the chemical space covered by the data sets, we performed Principal Component Analyses (PCAs) based on 13 physicochemical properties (see the Methods section) of the compounds and molecular similarity comparisons. The PCA plots shown in Figure 3a,b put the two data sets and the molecular diversity of approved drugs^36^ into perspective. From these plots, we conclude that the ChEMBL-derived data set is more focused on the chemical space most densely populated by the approved drugs, whereas the PubChem BioAssay-derived data set spreads more broadly across the chemical space of approved drugs. For both data sets, an accumulation of bioactive compounds in the area most populated by the approved drugs is apparent. The pairwise Tanimoto similarities of compounds in the two data sets, calculated using ECFP4 fingerprints, are compared in Figure 3c,d. The plots indicate that the active and inactive compounds from the ChEMBL and PubChem data sets are of moderate similarity. We further analyzed the intragroup similarity of active and inactive compounds within the ChEMBL and PubChem BioAssay-derived data sets. Although the PCA did not reveal clear distinctions in chemical features between active and inactive compounds, particularly within the drug-like range, Tanimoto similarity comparisons (Figure 3e,f) indicate lower similarity between the two classes, suggesting that machine learning models could effectively classify the compounds. The bioactive compounds in the ChEMBL-derived data set include 313 Murcko scaffolds,^37^ while the inactive compounds encompass 294 distinct scaffolds. The PubChem data set represents 146 scaffolds among the active compounds and 1,166 scaffolds within the inactive set. The most prominent Murcko scaffolds’ distribution within the ChEMBL and PubChem Bioassay-derived data sets are illustrated in Figure 3g,h. In the ChEMBL-derived data set, 26 Murcko scaffolds are represented by at least 10 compounds, while in the PubChem data set, 11 Murcko scaffolds meet this criterion (Table S1). The two bar charts further demonstrate that compounds with the same Murcko scaffold often have different activity classifications across data sets, especially for the ChEMBL data set. This allows the model to learn the importance of various derivatives beyond the primary scaffolds.

### Machine Learning Model Development and Validation

To build predictive models for aromatase inhibition, we trained four random forest (RF) classifiers selectively using the two data sets (ChEMBL and PubChem BioAssay) separately, with two types of molecular descriptors (208 physicochemical descriptors) and ECFP4 fingerprints (2048 bits), following the steps of cross-validation (CV), hyperparameter optimization, and model validation.

Before model building, the data sets were each split into a training set and a test set in ratio of 4:1. We performed 5-fold CV on the training set with similarity-based splitting and oversampling (see the Methods section: *Model development and validation*) with different hyperparameters, and recorded the best Boltzmann-enhanced discrimination of receiver operating characteristic (BEDROC) scores^38^—a metric used to evaluate the effectiveness of virtual screening methods in ranking active compounds early in a ranked list—of the average performance in 5-fold CV models and the corresponding hyperparameter (“max_features”). As shown in Table 2, the best model we obtained from the two data sets and descriptor combinations obtained BEDROC scores of 0.35 to 1.00. The high BEDROC scores of ChEMBL-based models indicate superior early enrichment. The PubChem-based models showed lower BEDROC values, likely due to the broader chemical diversity (Figure 3a,b) and data imbalance within the PubChem test set.

**Table 2:** Best Average BEDROC Scores Achieved during Five-Fold CV with Similarity-Based Splitting and Oversampling with the Selected max_features Value

model	BEDROC score	the max_features^a^ that contribute to best model performance
ChEMBL_ECFP4	1.00	0.20 (20%)
ChEMBL_RDKit 2D	1.00	0.80 (80%)
PubChem_ECFP4	0.35	Sqrt (square root of the total number)
PubChem_RDKit 2D	0.73	0.20 (20%)

a Maximum fraction of features considered per split.

**Figure 4:**
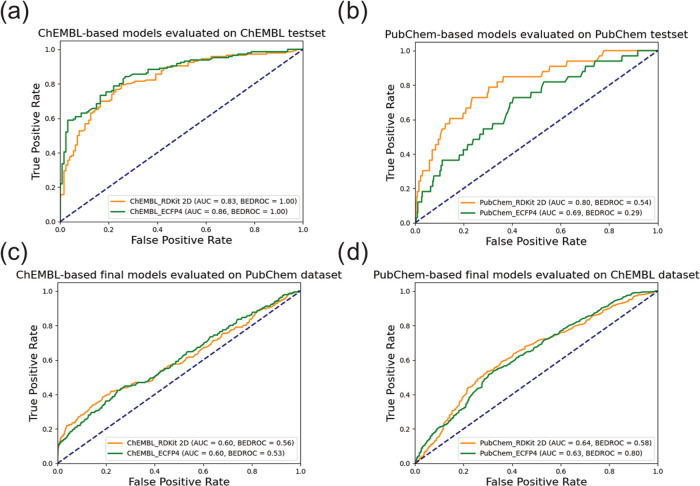
Receiver operating characteristic (ROC) comparison of (a) ChEMBL-based models evaluated on ChEMBL test set; (b) PubChem-based models evaluated on PubChem test set; (c) ChEMBL-based final models evaluated on PubChem data set; and (d) PubChem-based final models evaluated on ChEMBL data set.

The best hyperparameters identified through CV were used to train RF classifiers on the training sets, with oversampling applied to address the class imbalance. The RF classifiers were evaluated on the test set using receiver operating characteristic (ROC) curves (Figure 4a,b), with performance measured as the area under the ROC curve (ROC-AUC) and the BEDROC score (Table 3). The ROC-AUC values obtained by the four RF classifiers were between 0.69 and 0.86, whereas their BEDROC values ranged from 0.29 to 1.00. The BEDROC values revealed a discrepancy: while the ChEMBL-derived models performed well with strong early enrichment, the PubChem-derived models exhibited a lack of early enrichment ability. This might be due to artifacts arising from data imbalance and the smaller test set size of the PubChem data set (33 active data points), as BEDROC places significant weight on the top-ranked data points; in this case, the few top-ranked molecules dominate the BEDROC value.

**Table 3:** Performance of the Machine Learning Models

model	training data	feature set	test data	ROC-AUC	BEDROC
ChEMBL_train_ECFP4	ChEMBL training set	ECFP4 fingerprint	ChEMBL test set	0.86	1.00
ChEMBL_train_RDKit 2D	ChEMBL training set	RDKit 2D descriptors	ChEMBL test set	0.83	1.00
PubChem_train_ECFP4	PubChem training set	ECFP4 fingerprint	PubChem test set	0.69	0.29
PubChem_train_RDKit 2D	PubChem training set	RDKit 2D descriptors	PubChem test set	0.80	0.54
ChEMBL_all_ECFP4	ChEMBL data set	ECFP4 fingerprint	PubChem data set	0.60	0.53
ChEMBL_all_RDKit 2D	ChEMBL data set	RDKit 2D descriptors	PubChem data set	0.60	0.56
PubChem_all_ECFP4	PubChem data set	ECFP4 fingerprint	ChEMBL data set	0.63	0.80
PubChem_all_RDKit 2D	PubChem data set	RDKit 2D descriptors	ChEMBL data set	0.64	0.58

To maximize the use of the available data resources for the virtual screening campaign, the test sets were combined with the training sets to create a full-size data set for training the final models. This approach ensures that all available data contribute to model development, enhancing the predictive power and robustness of the resulting models. We also cross-validated the two data sets with the corresponding models (Figure 4c,d and Table 3). The ROC-AUC values were lower in this testing scenario, ranging from 0.60 to 0.64. The drop in performance compared to the test with data of the same origin is expected and consistent with common observations and reports. One important reason is that the two data sets cover distinct areas of the (drug-like) chemical spaces. Another major factor is differences in data annotation and data heterogeneity in general. Certainly, some—although not all—of these discrepancies were mitigated by following a binary classification rather than a regression approach.

Interestingly, the PubChem_ECFP4 model achieved a high BEDROC score when tested on the ChEMBL data set, which contrasts with its performance when trained on 80% of the PubChem data set and tested on the remaining 20%. This result highlights the model’s capacity to generalize across data sets despite its relatively low performance during internal cross-validation with the PubChem data set. This result led to the hypothesis that the active and inactive rate of data in the ChEMBL data set is more balanced than the PubChem test set, making it more suitable for validation with BEDROC. In addition, the PubChem_ECFP4 model may show bias against novel scaffolds from a different chemical space, leading to distinct performance when tested across data sets.

### Virtual Screening of Novel Inhibitors of Human CYP19A1

**Figure 5:**
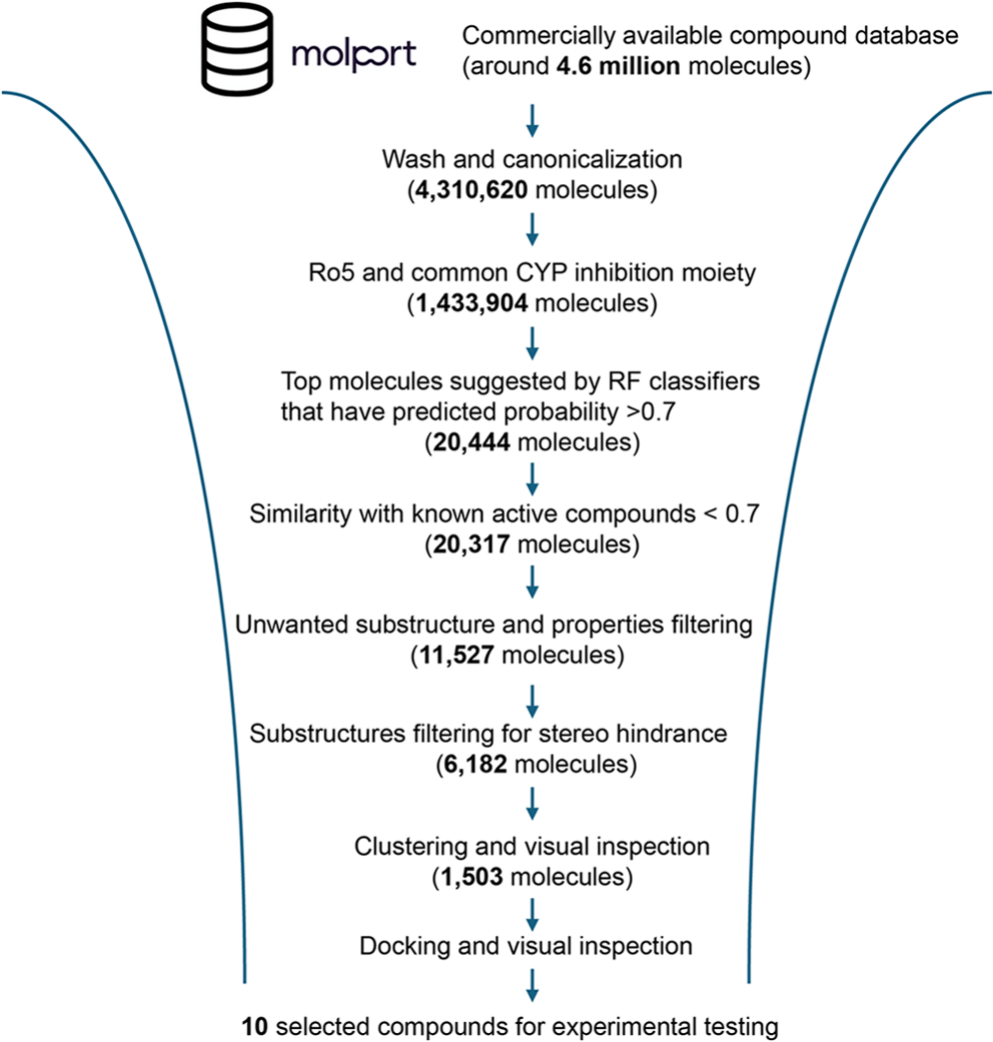
Schematic workflow of the virtual screening campaign, including the number of compounds remaining after each step.

To identify potential aromatase inhibitors, we employed the four trained models in a virtual screening campaign, prioritizing compounds for experimental validation based on their predicted activity, with the workflow shown in Figure 5. For this purpose, we used the MolPort database (www.molport.com), which contains approximately 4.6 million molecules. After applying a washing and canonicalization process (as detailed in the Methods section), a total of 4,310,620 molecules were retained for further analysis. The database was preprocessed by filtering for compounds that obey the rule of five (ro5)^39^ and the presence of substructures common to CYP inhibitors (imidazole, triazole, or pyridine), resulting in 1,434,904 molecules for screening. These molecules were then evaluated using the RF classifiers, which predicted the probability of a compound being a CYP19A1 inhibitor. Molecules ranked in the top 10,000 by each model and with a probability greater than 0.7 (20,444 molecules) were kept for further analysis. To ensure novelty, compounds with a similarity score greater than 0.7 to known active compounds in the ChEMBL and PubChem bioassay data sets were filtered out, narrowing the selection to 20,317 molecules. Further refinement involved the removal of undesirable substructures and properties, such as Pan-Assay Interference Compounds (PAINS),^40^ long alkanes and molecules with more than eight rotatable bonds, which reduced the pool to 11,527 molecules, followed by additional substructure filtering to avoid steric hindrance for CYP inhibition moieties, which yielded 6182 molecules. These molecules were subjected to similarity-based clustering followed by visual inspection to ensure the selection of chemically diverse hits, ultimately reducing the number to 1503. A final round of docking and visual inspection considering steric hindrance, shape complementary, and feasibility to reach the heme iron in the binding site led to the final selection of 10 promising compounds for experimental testing. The structures of the selected compounds are listed in Table 4, together with the structure of the most similar compound in the respective training set. The chemical space of the tested molecules was analyzed with the same dimensionality reduction using PCA as previously described, as shown in Figure 3i. The data points localized within the drug-like range, and also in the similar range of active data points of both data sets.

**Table 4:**
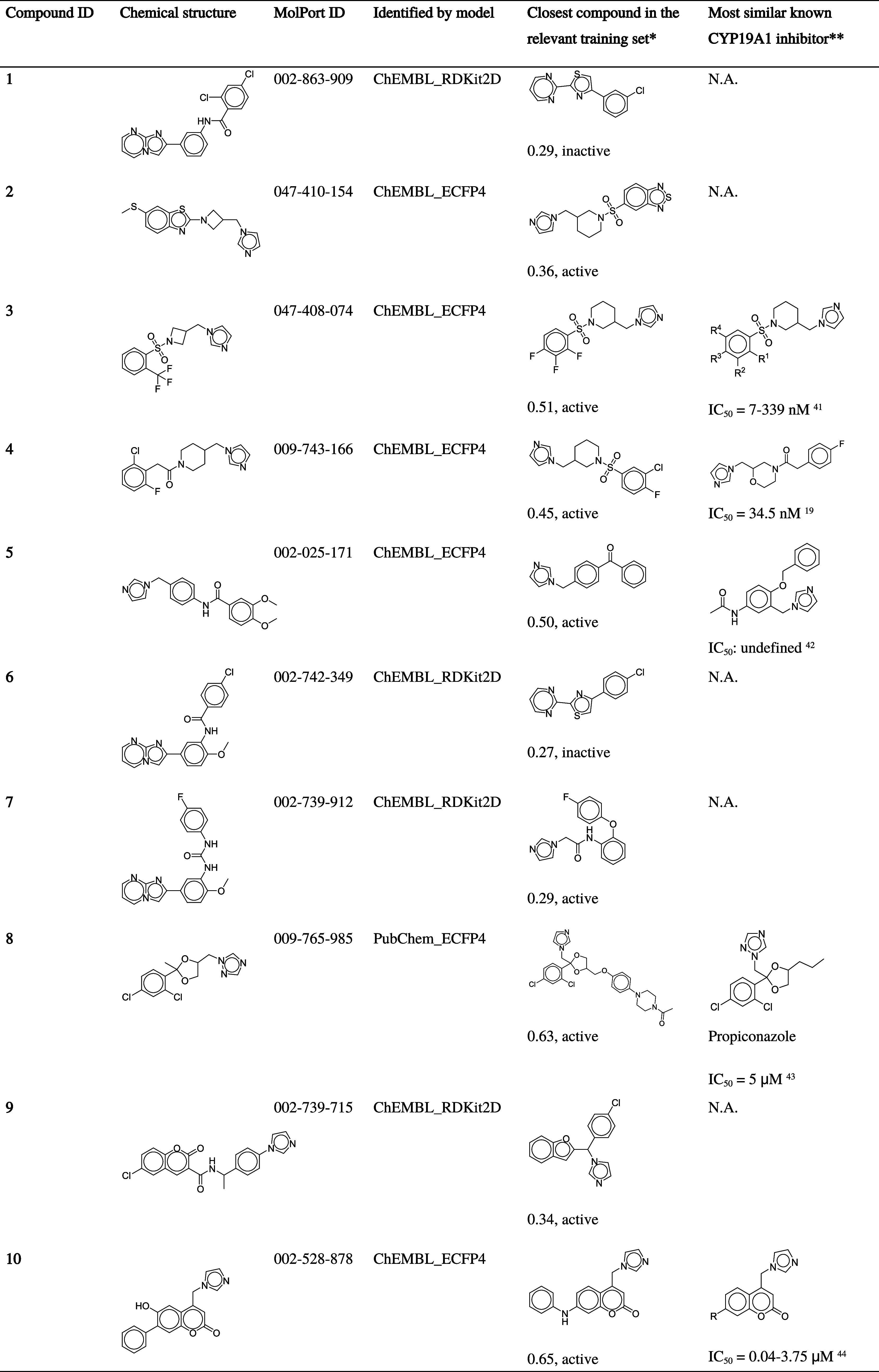
Aromatase Inhibitor Candidates Identified by Virtual Screening

* With additional information on the Tanimoto similarity compared with the inhibitor candidate, and activity label of the compound in the data set.

** According to a SciFinder search. The measured activity reported by literature was listed.^19,41−44^ N.A.: not available.

### In Vitro Inhibitor Testing of Candidate Compounds Suggested by Virtual Screening

**Figure 6:**
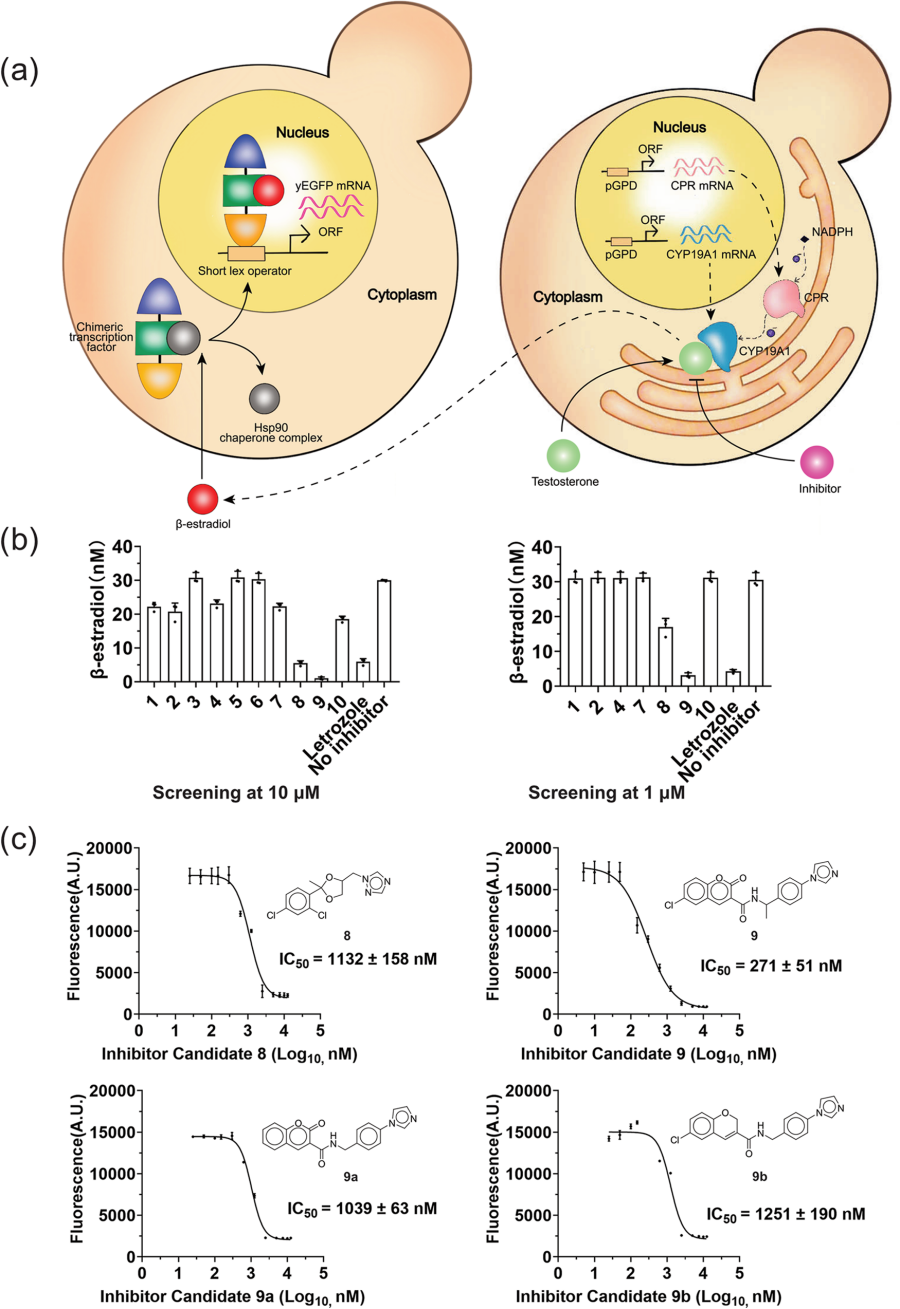
In vitro testing of CYP19A1 inhibitor candidates. (a) Illustration of the enzymatic assay based on heterologous expression of human CYP19A1 used for testing candidate CYP19A1 inhibitors. (b) Screening of compounds for human CYP19A1 inhibition by the 10 selected compounds and the reference compound letrozole at inhibitor concentrations of 10 μM (left panel) and 1 μM (right panel). (c) IC_50_ curves for **8**, **9**, **9a**, and **9b**. Data are presented as mean ± SEM (*n* = 3).

Two yeast strains were employed for screening and evaluating candidate compounds against the human CYP19A1 protein. One strain, engineered to express human CYP19A1 and cytochrome P450 reductase (CPR), served as an enzyme system (“enzyme bag”) capable of converting testosterone to β-estradiol. In the other strain, the β-estradiol then acts as an inducer, displacing an Hsp90 chaperone complex from a chimeric transcription factor that passes through the nuclear pore and binds to short lex operators. This binding enhances the expression of the reporter protein yEGFP (yeast-enhanced green fluorescent protein), as illustrated in Figure 6a. The corresponding fluorescence signals can then be monitored by flow cytometry. When inhibitors are added together with the aromatase substrate testosterone, the enzymatic activity of human CYP19A1 decreases, leading to reduced β-estradiol production. In turn, a lower fluorescence signal is observed.

Ten compounds identified through virtual screening were tested in this aromatase inhibitor screening assay to evaluate their relative potency compared to letrozole.^45^ Initially, these inhibitors were applied at a final concentration of 10 μM. Compounds **1**, **2**, **4**, **7**, **8**, and **9** demonstrated significant inhibition of CYP19A1 activity at 10 μM and were further tested at 1 μM. At this concentration, only **8** and **9** significantly inhibited CYP19A1 activity (Figure 6b). Among these two, the imidazole and coumarin ring containing **9** (*N*-(1-(4-(1*H*-imidazol-1-yl)phenyl)ethyl)-6-chloro-2-oxo-2*H*-chromene-3-carboxamide) was identified as the most effective inhibitor of CYP19A1, with an inhibition rate of 13 ± 2%, as compared to 17 ± 1% for letrozole (Table S2). As **9** was received as a racemate and exhibited potent CYP19A1 inhibition, two analogs of **9** (**9a** and **9b**), lacking the methyl group at the stereocenter, were identified from the Enamine database. In addition, **9a** lacks a Cl atom and **9b** lacks a keto group in comparison to **9**. These analogs, along with **8** and **9**, were also tested for their IC_50_ values (Table 5 and Figure 6c). Again, **9** proved to be the most potent inhibitor, with an IC_50_ value of 271 nM.

**Table 5:** Measured IC_50_ Value of Compounds **8**, **9**, **9a**, and **9b**

compound name	IC_50_ value (nM)
**8**	1132 ± 158
**9**	271 ± 51
**9a**	1039 ± 63
**9b**	1251 ± 190

Several factors were considered to avoid assay artifacts.^46^ The biosensor gene circuit used in this study is orthogonal to the yeast chassis,^47^ ensuring no interference between the biosensor and the yeast host, thus preventing false positives. Furthermore, the concentrations of β-estradiol used in the tests were demonstrated to be noncytotoxic, which is crucial in avoiding both false-positive and false-negative results.^48^ To further confirm the absence of interference between the inhibitors and the biosensor, a control test was conducted, where a 10 μM inhibitor sample was incubated with the biosensor in the presence of 30 nM testosterone and 30 nM β-estradiol. The results showed no significant difference from the control group (without the inhibitor), as shown in Figure S1.

### Computational Analysis of the Hit Compounds

**Figure 7:**
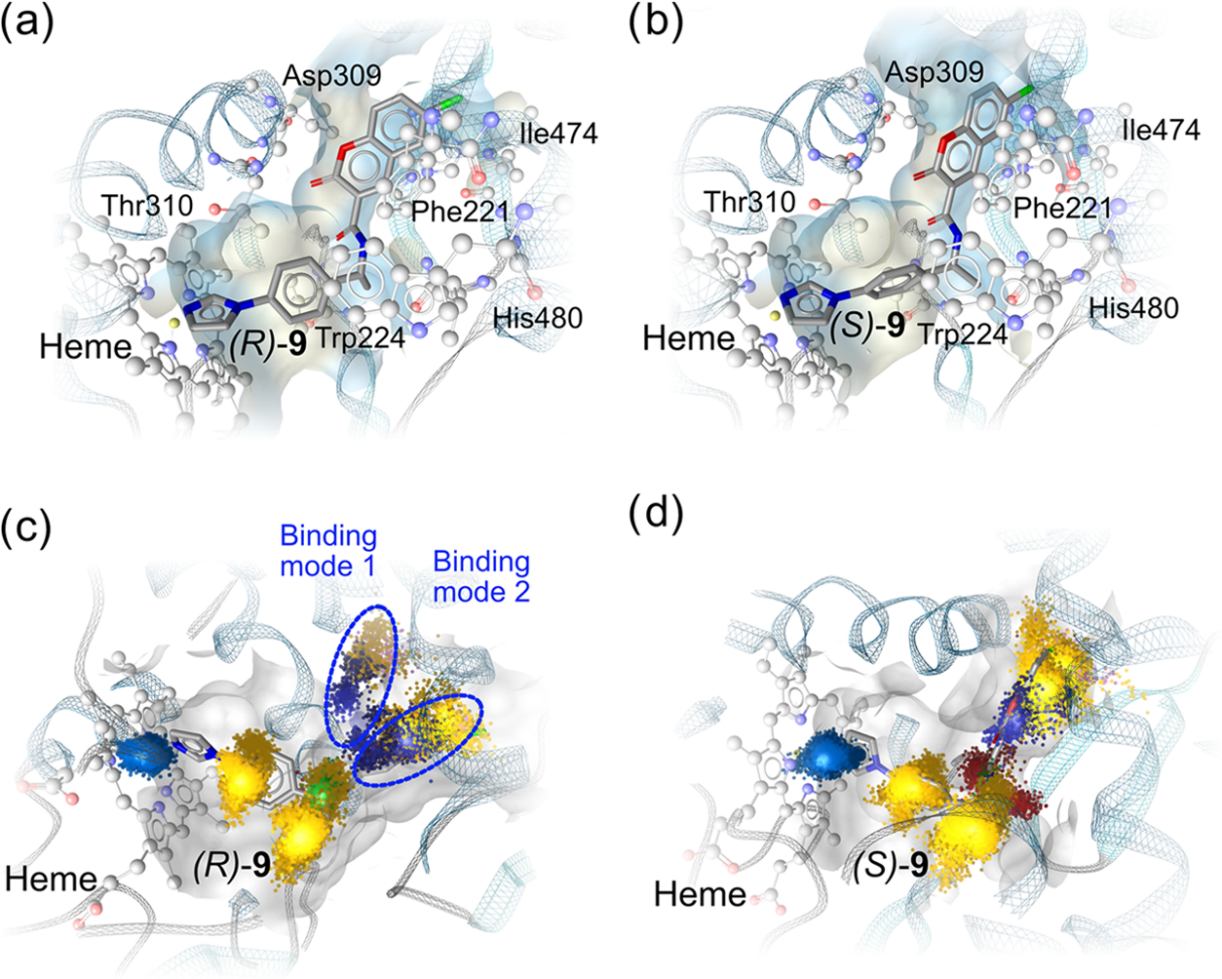
(a, b) Docking poses of *(R)***-9** and *(S)*-**9** shown in the CYP19A1 binding site. (c, d) Dynamic pharmacophore (dynophore) interactions shown as interaction clouds showing the spatial extent of *(R)***-9** and *(S)*-**9** binding to the CYP19A1 active site during MD simulations. Iron binding is shown as light blue clouds, hydrophobic contacts are shown as yellow cloud, hydrogen bond donors (HBD) are shown as green clouds, hydrogen bond donors (HBA) are shown as red clouds, aromatic interactions are shown as dark blue clouds, and halogen bond interactions are shown as purple clouds.

The most active inhibitor of CYP19A1 in this study, an imidazole and coumarin ring-containing molecule, **9**, possesses a single stereocenter and was purchasable only as a racemic mixture. To further understand the stability of the interactions and the binding mode in a dynamic system, both *(R)-***9** and *(S)-***9** were docked to the CYP19A1 binding site (Figure 7a,b), and these bound conformations served as starting points for MD simulations. Both isomers consistently inhibited the heme iron during our MD simulations of 100 ns in five replicas: in 99.4% of the trajectories of *(S)-***9** and 100% for *(R)-***9**. The hydrophobic contacts between both isomers’ imidazole-connected benzene ring and the nearby lipophilic residues (i.e., Ile133, Trp224, Thr310 and Val310) in the binding site of CYP19A1 were observed in more than 99% of the trajectories. The hydrophobic contacts between both isomers’ methyl groups at the stereo centers and Leu477, Trp224 and Phe134 of CYP19A1 were also stable during the simulations (100% of trajectories for *(S)-***9** and 99.8% of trajectories for *(R)-***9**). We also compared **9a** and **9b** with the same MD simulation settings. The absence of the methyl group did not cause any decrease in heme inhibition, stressing the importance of the overall shape complementary of the protein with the molecule rather than the difference of a single methyl group at the stereocenter. Interestingly, *(S)*-**9** exhibited an additional interaction between its carbonyl oxygen and Ser478 in 19.9% of the trajectories, which was absent in *(R)*-**9**. We visualized the interaction between **9** and CYP19A1 using our in-house dynamic pharmacophore (dynophore) tool,^49,50^ which summarizes the interactions during MD simulations in each trajectory. Interestingly, we found two plausible binding modes for *(R)-***9** and one for *(S)-***9**, as shown in Figure 7c,d. The binding modes 1 and 2 of *(R)-***9** mainly differ in the positioning of the interactions contributed by the coumarin moiety. The interaction patterns of the two binding modes are similar—π–π interactions between the 2-pyrones and Phe221 or His480, π–π interactions between the coumarin benzene ring and His480, and hydrophobic contacts between the coumarin benzene ring and Phe221. In addition, a halogen bond interaction between the chlorine and Arg192 was observed during 4% time of the MD simulation while the molecule is arranged in binding mode one, and hydrophobic contacts with the chlorine group with Phe221 in binding mode 2. The two binding conformations majorly differ due to protein flexibility but not different interactions. For *(S)-***9**, the coumarin moiety was stabilized through aromatic interactions with Phe221, and hydrophobic contacts with Phe221 and Val313.

During our analysis of the binding mode, we hypothesized that either Asp309 or Ser478 could potentially be targeted by **9** as a covalent binder. For Asp309 to serve as a viable target, it must be protonated. There are reported cases in the literature where aspartic acid residues have been successfully targeted by covalent binders, highlighting the feasibility of such an approach in drug design.^51^ In our MD simulation setup, Asp309 was automatically calculated to be protonated, based on the microenvironment of the binding site. The crystal structures also suggest that protonation of Asp309 is necessary to stabilize a water molecule involved in steroidal transformation.^16,52^ With respect to 9, its aromatic 2-pyrone ring enables electron delocalization toward the α,β-unsaturated carbonyl structure. When linked via the amide bond, this structural feature suggests that the compound could function as a weak covalent binder, potentially acting as a Michael acceptor. Ring-opening reactions of 2-pyrones have been observed in nature.^53^ After the hypothetical reaction (Figure S2), the resulting carboxylic acid carboxylate could form an ionic bond with His480, while the remaining benzene ring could maintain π–π interactions. However, no significant differences were detected when we tested this hypothesis by comparing the activity of compound **9** with and without preincubation during culturing. Our inhibitor occupies the binding pocket that exemestane (PDB code: 3S7S)^16^ faces to establish the covalent bond, as compared in Figure S3. The residues potentially targeted by the warhead of exemestane are Asp309 or Ser478. **9** does not seem to target these residues as seen in the assay. Despite being close to Asp309 or Ser478, **9** is far away from heme iron, making it unlikely to undergo the same inhibition reaction as exemestane.

## Conclusions

The conformational flexibility and complex coordination chemistry of CYP19A1 pose formidable challenges to structure-based methods such as docking. In view of these challenges, we explored the capacity of ML to learn the existing measured data on CYP19A1 inhibition and identify novel, promising CYP19A1 blockers from libraries of purchasable compounds. By training the ML models on two distinct data sets (derived from the ChEMBL database and PubChem BioAssay), our ML approach could cover a vast chemical space. Using BEDROC as the primary performance metric, we obtained models yielding sound early enrichment, which is key to the success of screening campaigns.

Harnessing the ML models, established compound filters, and clustering, we reduced a library of commercially available compounds from 4.6 million to 1500 compounds. From these 1500 compounds, we selected ten compounds for experimental validation, of which seven showed activity in an enzymic assay.

ML accelerates the early filtering stage in virtual screening campaigns, which were traditionally performed using high-throughput docking for other targets but are less feasible for CYPs, and docking, combined with visual inspection, complements this process by incorporating structure-based knowledge into the final selection for the success of our screening process.

With an IC_50_ value of 271 ± 51 nM, we identified compound **9** as the most potent inhibitor of CYP19A1 among the ten selected compounds. Interestingly, the compound, containing an imidazole moiety and a coumarin ring connected as a different scaffold, is structurally clearly distinct from any known NSAIs. We also derived the likely binding mode of **9** interacting with CYP19A1 by utilizing structure-based modeling approaches and MD simulations. We hope these insights and results will support the further development of much-needed, innovative inhibitors of CYP19A1.

## Methods

### Data Set Preparation and Analysis

Two separate data sources were utilized to investigate compound activity against CYP19A1 (*Homo sapiens*). The first source was the ChEMBL database^33^ (Target ID: CHEMBL1987, accessed March 2023). From this database, compounds with reported IC_50_ values and corresponding pChEMBL values were selected. Compounds with a pChEMBL value ≥6 were categorized as active, while those with lower values were categorized as inactive. The second source was PubChem^32^ BioAssay 743139 (Tox21), which summarizes data from BioAssay 743083 (aromatase antagonist mode assay) and BioAssay 7431084 (cell viability counter assay), both targeting CYP19A1. Compounds were labeled as inactive if their PUBCHEM_ACTIVITY_SCORE was 0, active if the score ranged between 40 and 100, and those with scores between 5 and 30 were excluded from the study. The data sets from the ChEMBL database and PubChem BioAssay database were analyzed separately and were not combined for model training.

All compounds from both data sources were standardized using the ChEMBL Structure Pipeline.^54^ Additional standardization steps were applied, including tautomer canonicalization, removal of stereoisomer information, and elimination of salt components from SMILES strings according to the rules described in Hit Dexter.^55^ Compounds containing elements other than the defined common organic compounds elements (i.e., H, B, C, N, O, F, Si, P, S, Cl, Se, Br, and I) or with molecular weights outside the range of 250–900 Da were excluded. These preprocessing steps were carried out using the “csp_wash” method from the “MoleculePreprocessorExtended” class in the RingSystems library.^56^ Additionally, duplicates within each data set were removed based on canonical SMILES. If compounds in the same data set had identical labels, only one was retained, while compounds with conflicting labels were removed. When checking the ChEMBL and PubChem data sets for inconsistently classified compounds, we identified a single compound, nordihydroguaiaretic acid, which were labeled differently in the two data sets. In the ChEMBL data set, the activity data was curated from the summary of a virtual screening study, reporting an IC_50_ value of 11 nM. However, upon tracing back to the original study cited by this virtual screening work in 1993, the compound was reported with an activity of 11 μM.^57^ To ensure consistency in data treatment across the entire data set, we retained the data point as it appeared in the ChEMBL data set, as it would not significantly impact the overall performance of the model.

DrugBank^36^ (download date: March 14th, 2023) was preprocessed using the same workflow as the ChEMBL and PubChem data sets and subsequently used for chemical space comparison. The prepared data sets were analyzed via principal component analysis (PCA), which was based on 13 physicochemical properties of the compounds calculated with RDKit (version 2021.03.2):^58^ number of nitrogen atoms, number of oxygen atoms, number of chiral centers, molecular weight, number of heavy atoms, number of hydrogen bond acceptors, number of hydrogen bond donors, log *P*, topological polar surface area, number of aromatic atoms, sum formal charge, number of rings, and the fraction of sp3 hybridized carbon atoms.

### Model Development and Validation

The molecular structures were featurized using two types of molecular descriptors: RDKit 2D descriptors (208 physicochemical descriptors) calculated with “MolecularDescriptorCalculator” function and ECFP4 fingerprints (2048 bits) calculated with “GetMorganFingerprintAsBitVect” function from RDKit.^58^ The ChEMBL and PubChem BioAssay data sets were each randomly split into a training set and an external test set using the “train_test_split” method from scikit-learn (version 1.3.2),^59^ with a 4:1 train-test ratio, shuffling enabled, and the random_state set to 42.

In total, four Random Forest (RF) classifiers were trained using the ChEMBL and PubChem BioAssay data sets, with either RDKit 2D descriptors or ECFP4 fingerprints. The RF classifiers were developed using the scikit-learn library, with the following fixed hyperparameters: “n_estimators” was set to 1000 to build a robust ensemble of decision trees, “min_samples_split” was set to 2 to allow more flexible splits with minimal samples, and the “random_state” was fixed at 42 to ensure reproducibility. In addition, “max_features” was optimized during cross-validation exploring values of None, “sqrt”, 0.2, 0.4, and 0.8 to control the number of features considered for each split. The optimal parameters were selected based on maximizing the Boltzmann-enhanced discrimination of receiver operating characteristic (BEDROC).^38^

To ensure the model’s ability to predict novel structures, compounds with a Tanimoto similarity (calculated by “BulkTanimotoSimilarity” function from RDKit) greater than 0.3 were kept separate in the training and testing splits during cross-validation: the training sets, as described in the Data Set Preparation and Analysis section, were clustered based on the similarity using Butina clustering algorithms,^60^ allowing lowest Tanimoto score of 0.3. The resulting clusters were shuffled and distributed into five groups, with the principle of keeping the total number of compounds in each group as similar as possible. Followed by clustering, to address class imbalance, we employed Synthetic Minority Oversampling Technique (SMOTE) or Synthetic Minority Oversampling Technique for Nominal and Continuous data (SMOTENC) from the Imbalanced-learn library^61^ to the training data within each fold during cross-validation (CV) with the steps of: 1. Split the data into training and test sets for the current fold. 2. Apply SMOTE to the training set to balance the class distribution. 3. Train the classifier using the oversampled training set. 4. Predict probabilities for the test set using the trained classifier. 5. Record predictions for performance evaluation.

The optimum hyperparameters were used to train the RF classifiers on the whole training set, with same oversampling method applied for both training models on the training data for external testing, and models on the complete data for screening. The performance was evaluated on the external test set using metrics such as the area under the receiver operating characteristic curve (AUC-ROC) and BEDROC. After evaluation, each test set was combined with the corresponding training set to develop the final model, which was then applied for virtual screening.

### Virtual Screening

All stock compounds from MolPort (www.molport.com) were retrieved (downloaded date: March 10th 2021) and used for virtual screening. The same standardization steps used for the ChEMBL and PubChem BioAssay data sets were applied to the MolPort compounds. The database was also preprocessed by filtering for compounds that obey the rule of five (ro5) and the presence of the common substructure of CYP inhibitors (imidazole, triazole, or pyridine). ECFP4 and RDKit 2D descriptors were calculated for all the remaining compounds in the database. These calculated descriptors were then used as input for the trained RF classifiers, and the predicted probabilities of the compound being active were assigned from each model.

During virtual screening, several filters and criteria were applied to refine the compound selection. Compounds were retained if their predicted probabilities exceeded 0.7 and were ranked among the top 10,000 by any of the four models. To ensure novelty, only compounds with similarity to any known active compounds below 0.7 were included. Compounds matching any PAINS pattern were excluded, as were compounds with specific undesirable features such as a positive charge, long alkane moieties, bromine or iodine groups, nitro groups, or basic amine groups. Additionally, compounds with fewer than eight rotatable bonds were retained to prioritize those with favorable conformational flexibility.

The remaining compounds were clustered with a maximum Tanimoto similarity of 0.6 based on ECFP4 fingerprints (2048 bits), and compounds of interest from different clusters were manually selected for further analysis. The selected compounds were docked to assess potential steric hindrance and their ability to reach the heme iron in the binding site.

### Protein Atomistic Structure Preparation

The atomistic structure of human CYP19A1 was obtained from the Protein Data Bank^62^ (PDB entry: 5jkv(15)). Co-crystallized water molecules, androstenedione, pentaethylene glycol, and phosphate ions were removed, while the protein structure, including the heme prosthetic group, was retained. The structure was prepared using MOE v.2020.0901 (Molecular Operating Environment; Chemical Computing Group ULC, Montreal, QC, Canada), utilizing the integrated Structure Preparation tools. The structure was optimized and protonated using Protonate3D^63^ with the OPLS-AA^64^ force field.

### Molecular Docking

Selected small molecules were docked into the binding pocket of the prepared CYP19A1 structure using GOLD v.5.8.1 (Genetic Optimization for Ligand Docking; CCDC Software, Cambridge, U.K.).^65^ The search efficiency was set to 100%, generating 10 docking poses per molecule. The docking center was defined by the coordinates of the heme iron, with a surrounding sphere of 10 Å radius. The scoring function used was “goldscore_p450_cds”, while all other settings remained at their default values. The docking results were visualized using LigandScout v.4.4.3,^66,67^ and the conformations were further minimized with the MMFF94 force field.^68^

### Molecular Dynamics (MD) Simulation and Dynamic Pharmacophore (Dynophore) Generation

In order to investigate the interaction dynamics of **9** and its analogs to CYP19A1, all-atom MD simulations were carried out with the most plausible binding hypotheses as starting conformation. The prepared complex was loaded into Maestro v. 13.1.137 (Schrödinger Release 2022–1: Maestro, Schrödinger, LLC, New York, NY) for structure optimization with the implemented functionality “Protein Preparation Wizard”. The heme iron atom type was manually corrected to Fe^3+^. The simulation environment was prepared with “System Builder”. The protein–ligand structures were solvated in cubic water boxes with padding of 10 Å filled with TIP3P water model.^69^ Automatically calculated numbers of chloride or sodium ions were added to each system for neutralization, and another 0.15 M NaCl was added to mimic the physiological environment. The generated systems were simulated with the Desmond simulation engine v. 6.9^70^ on water-cooled NVIDIA RTX 2080 Ti graphical processing units (GPU) for 100 ns in five replicas for each system, using the OPLS-AA force field. The simulation temperature and pressure were kept at their default values of 300 K and 1.01325 bar, respectively. The system was relaxed and equilibrated following the standard seven-step protocol. System coordinates were recorded every 100 ps. The coordinate and trajectory files were wrapped and aligned with VMD v. 1.9.3.^71^ For the full simulation trajectory, the frequencies of inhibitor-enzyme interactions were derived and analyzed using the dynamic three-dimensional (3D) pharmacophores analysis method (“dynophores”).^49,50^

### Chemical and Reagents

The inhibitor candidates (**1**–**10**) were purchased from MolPort (Riga, Latvia). Compound **1** was obtained from BIONET—Key Organics Ltd., compounds **2**–**4** were obtained from Life Chemicals Inc., and compounds **5**–**10** were obtained from Vitas M Chemical Limited. Compounds **9a** and **9b** were purchased from Enamine. Letrozole was obtained from Enamine Ltd. (Riga, Latvia).

Compounds **1**–**10, 9a, 9b** and letrozole were >95% pure based on HPLC analysis, as listed in the Supporting Information. The chromatographic analysis was performed using an Agilent 1290 Infinity HPLC system, consisting of a binary pump, autosampler, and column compartment, coupled with an Agilent 1260 DAD VL+ Detector and an Agilent 6130B Single Quadrupole MS. Separation was achieved on an Agilent Poroshell C18 column (100 × 2.1 mm^2^, 2.7 μm particle size) maintained at 30 °C. The mobile phase comprised Solvent A (Water with 0.1% Formic Acid) and Solvent B (Acetonitrile with 0.1% Formic Acid), delivered at a flow rate of 0.400 mL/min with the following gradient: 5% B from 0.0 to 1.0 min, linearly increased to 95% B from 1.0 to 8.0 min, held at 95% B until 10.0 min, then returned to 5% B by 10.5 min, with a total runtime of 11.00 min. The injection volume was 0.5 μL. The DAD monitored signals at 254, 210, and 220 nm (with the latter used for analysis), at a scan rate of 40 Hz. The MS operated in both positive and negative scan modes, with an *m*/*z* range of 50–700.

Synthetic defined complete (SDC) liquid medium was used as the culture medium of *Saccharomyces cerevisiae*. The medium was composed of 79.2 mg histidine (H108260 Aladdin Beijing, CN), 396 mg leucine (L104898 Aladdin Beijing, CN), 79.2 mg tryptophan (T103480 Aladdin Beijing, CN), 79.2 mg uracil (U102087 Aladdin Beijing, CN), 2 g amino acid mix (0.5 g adenine, and 2.0 of each of the following amino acids: adenine, alanine, arginine, asparagine, aspartic acid, cysteine, glutamine, glutamic acid, glycine, isoleucine, lysine, methionine, proline, serine, threonine, tyrosine, valine, phenylalanine—Aladdin Beijing, CN), 6.7 g Yeast Nitrogen Base (Q30009 Thermo Fisher Beijing, CN) and 20 g glucose (G116307 Aladdin Beijing, CN). These components were dissolved with 1 L double distilled water and autoclaved to prepare the SDC liquid medium. Steroids β-estradiol (E2758) and testosterone (T5411) were purchased from Merck (Beijing, CN).

### Flow Cytometry Assay

The biosensor strains, initially obtained from synthetic selective medium plates (diluted to an OD_600_ of 0.04), were precultured in 3 mL of SDC liquid medium at 30 °C and 240 rpm for 14 h. Then, 20 μL of the precultured biosensor cell solution (diluted to an OD_600_ of 5.00) was added to 1980 μL of SDC medium containing β-estradiol, testosterone, and an inhibitor. This 2 mL solution, containing the inducer and biosensor cells, was incubated at 30 °C and 240 rpm for 24 h. After incubation, 5 μL of this cell suspension was diluted into 195 μL of double-distilled water (1:40 dilution) before analysis using a BD FACSVerse flow cytometer (laser 488 nm, FITC filter 527/32 nm). A total of 10,000 cell events were collected per measurement. Performance Quality Control (PQC) was conducted monthly, with fluorescent beads (BD FACSuite CS&T Research Beads 650621) used to adjust the FITC voltage, ensuring reliable and reproducible FACS results. The relative difference between bead peaks was required to be less than 5% when comparing each measurement to the initial bead measurement. The *flowCore* R-Bioconductor package was used for the analysis of the FACS data, with each mean value calculated from flow cytometry measurements of three independent experiments.

### Aromatase Inhibitors Screening Assay

The *S. cerevisiae* strain byMM1712, which expresses both human cytochrome P450 reductase (CPR) and human CYP19A1, was incubated in 10 mL of Synthetic Defined Complete (SDC) liquid medium at 30 °C and 240 rpm for 14 h. A 14-h precultivation period ensured that the aromatase catalytic capacity remained consistent across all independent experiments and the inhibitor cannot act on P450 expression levels. The initial OD_600_ was diluted to 0.04, and then the yeast culture’s OD_600_ was measured and adjusted to 2.00 with fresh SDC medium. One mL of yeast solution (OD_600_ = 2.00) was poured into the wells of a 48-well deep-well cell culture plate (Axygen P-5 ML-48-C). Subsequently, 1 mL of SDC medium containing 120 nM testosterone and either 10 μM or 1 μM of the inhibitor was added to each well containing the yeast solution. The mixture was incubated at 30 °C and 240 rpm for 2 h. After incubation, 1.5 mL of the mixture from each well was transferred to 2 mL tubes and centrifuged at 16,000 rpm to pellet the yeast cells. Then, 1.2 mL of the supernatant was moved to 1.5 mL tubes and labeled according to the inhibitor concentration. For each sample, 1 mL of the supernatant was transferred to a different well of a 48-well deep-well plate. Next, 980 μL of SDC medium and 20 μL of the precultured byMM1984 biosensor cell solution (OD_600_ adjusted to 5.00 after 14 h of preculture) were added to each well. Finally, flow cytometry was performed as previously described. The entire assay was repeated in three independent experiments to calculate the mean values.

### Statistical Analysis

All repeated experimental results are presented as mean ± SD or mean ± SEM. Statistical significance was determined using a two-tailed *t* test. Differences were considered significant if *P* < 0.05. IC_50_ curve was obtained by fitting the raw data to the empirical Hill function, where y is fluorescence (A.U.), and x is proportional to the inhibitor concentration (log_10_, nM). Statistical analysis was done with GraphPad Prism 8.01 (GraphPad Software Inc., La Jolla, CA). All repeated experimental results are presented as mean ± SD or mean ± SEM. Statistical significance was determined using a two-tailed *t* test, with differences considered significant if *P* < 0.05. The IC_50_ curve was generated by fitting the raw data to the empirical Hill equation, where *y* represents fluorescence (A.U.), and *x* is the log10-transformed inhibitor concentration (nM). Statistical analyses were performed using GraphPad Prism 8.01 (GraphPad Software Inc., La Jolla, CA).

## Data Availability

The source code for the model and the Molecular Dynamics input files, parameter files, topology files, analysis scripts can be found at https://github.com/sijie-liu97/CYP19_inhibitor_screening.

## Supporting Information Available

HPLC-MS report (PDF)
Model of human CYP19A1, obtained from the Protein Data Bank (PDB entry: 5jkv) with a docking pose of (*R*)-**9** (PDB)
Model of human CYP19A1, obtained from the Protein Data Bank (PDB entry: 5jkv) with a docking pose of (*S*)-**9** (PDB)
Molecular formula strings (TXT)
Corresponding SMILES and number of active/inactive compounds of the Murcko Scaffolds in the ChEMBL/PubChem data set; relative potency of candidate inhibitors in comparison to letrozole; control test for confirming the absence of interference between the inhibitors and the biosensor; hypothesis and experimental insight of whether compound **9** covalently binds to human CYP19A1; and comparison of (*R*)-9 and exemestane in the CYP19A1 active site (PDF)

## Supplementary Material

ci5c00204_si_001.pdf

ci5c00204_si_002.pdb

ci5c00204_si_003.pdb

## Notes

**Declaration of Generative AI and AI-Assisted Technologies in the Writing Process** While preparing this work, the authors used ChatGPT to improve the manuscript’s readability. After using this tool/service, the authors reviewed and edited the content as needed and take full responsibility for the publication’s content.

## Abbreviations

AUC-ROC: area under the receiver operating characteristic curve
BEDROC: Boltzmann-enhanced discrimination of receiver operating characteristic
CADD: Computer-Aided Drug Design
CYP: cytochrome P450
ER: estrogen receptor
IC_50_: half-maximal inhibitory concentration
PCA: principal component analysis
RF: random forests
