# Supplementary material for: Integrated Virtual Screening Approach Identifies New CYP19A1 Inhibitors
Source: J Chem Inf Model. 2025 Mar 19;65(7):3529–43. doi: 10.1021/acs.jcim.5c00204 (PMC12004523; doi:10.1021/acs.jcim.5c00204)
Supplement: Supplementary file 1 — ci5c00204_si_001.pdf [file ci5c00204_si_001.pdf]

Analysis Report

Agilent | Trusted Answers

Sample Information

**Name**	1	**Structure**	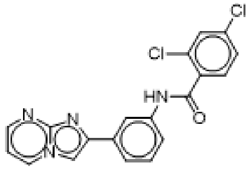
**Sample ID**			
**Instrument**	LC-SQ		
**MS Type**	Q		
**Inj. Vol. (ul)**	-1		
**Position**	P1-A2		
**Plate Pos.**		**MW**	383.32
**Operator**	SYSTEM	**Result Summary**	

Sample Chromatograms

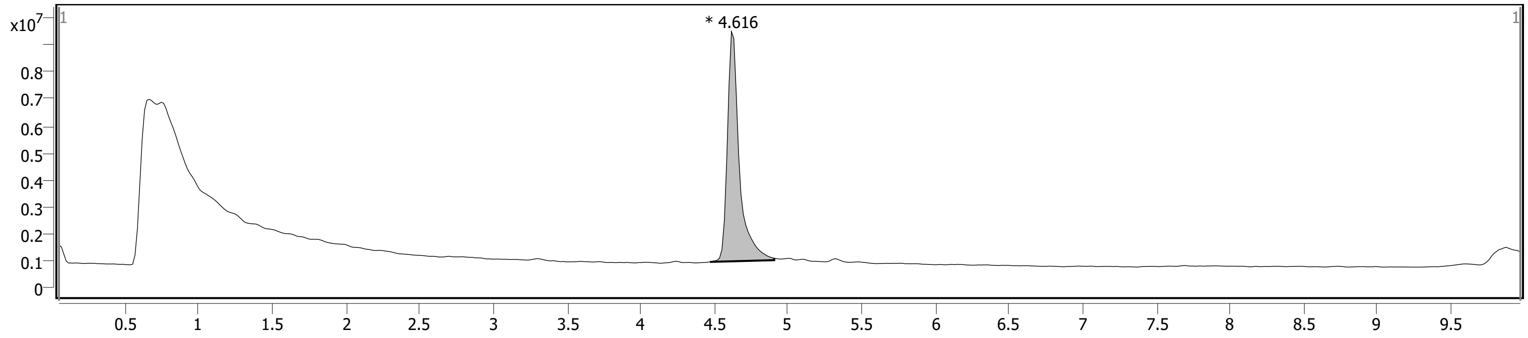

Chromatogram Peaks

Peak	Start	RT	End	Height	Area	Area %	SNR
1	4.470	4.616	4.906	8536232	48572090	100.00	

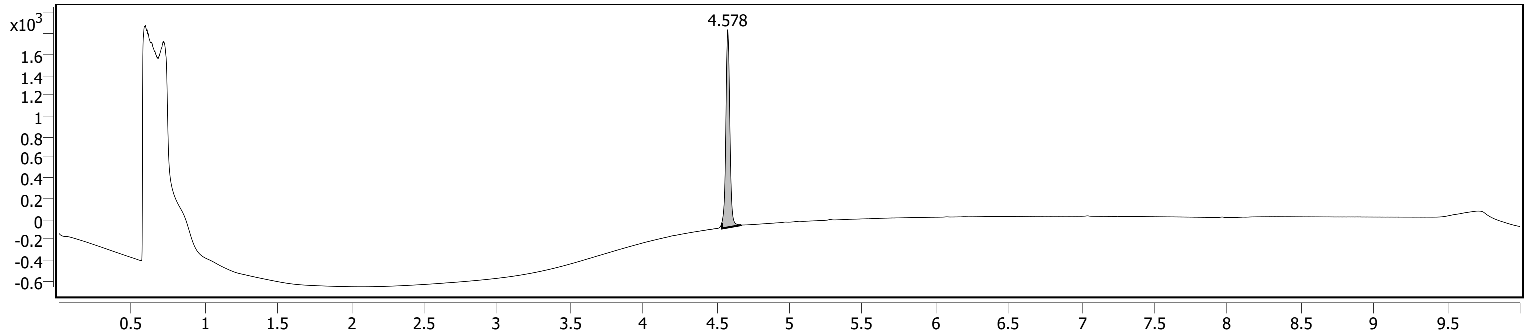

Chromatogram Peaks

Peak	Start	RT	End	Height	Area	Area %	SNR
1	4.538	4.578	4.671	1922	3688		

Sample Spectra

+ Scan (rt: 4.567-4.745 min) **Peak 1 from + TIC Scan**

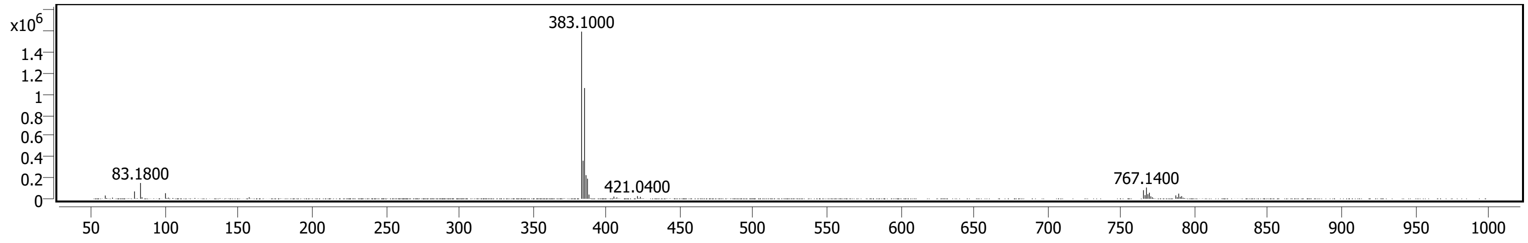

Spectrum Peaks

m/z	Z	Abund	Abund %	m/z (Calc)	Diff (ppm)	Ion Species	Formula	Ion Type
59.2000		31925	2.00					
79.1000		70860	4.44					
83.1800		151179	9.47					
100.1500		53667	3.36					
383.1000	1	1596587	100.00					
384.1000	1	363931	22.79					
385.1000	1	1057899	66.26					
386.1000	1	226214	14.17					
387.1000	1	192575	12.06					
388.1000	1	40859	2.56					
405.0500		21367	1.34					
421.0400		26078	1.63					
423.0400		19036	1.19					
765.1000	1	82548	5.17					
766.1900	1	35244	2.21					
767.1400	1	108031	6.77					
768.1200	1	44452	2.78					
769.1100	1	58366	3.66					
770.1300	1	23679	1.48					
787.1000		32882	2.06					
789.1000	1	49325	3.09					
790.1000	1	19403	1.22					
791.1000	1	25518	1.60					

MassHunter Qual 10.0
(End of Report)

Analysis Report

Agilent | Trusted Answers

Sample Information

**Name**	2	**Structure**	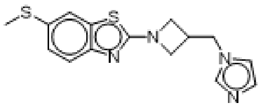
**Sample ID**			
**Instrument**	LC-SQ		
**MS Type**	Q		
**Inj. Vol. (ul)**	-1		
**Position**	P1-A6		
**Plate Pos.**		**MW**	316.44
**Operator**	SYSTEM	**Result Summary**	

Sample Chromatograms

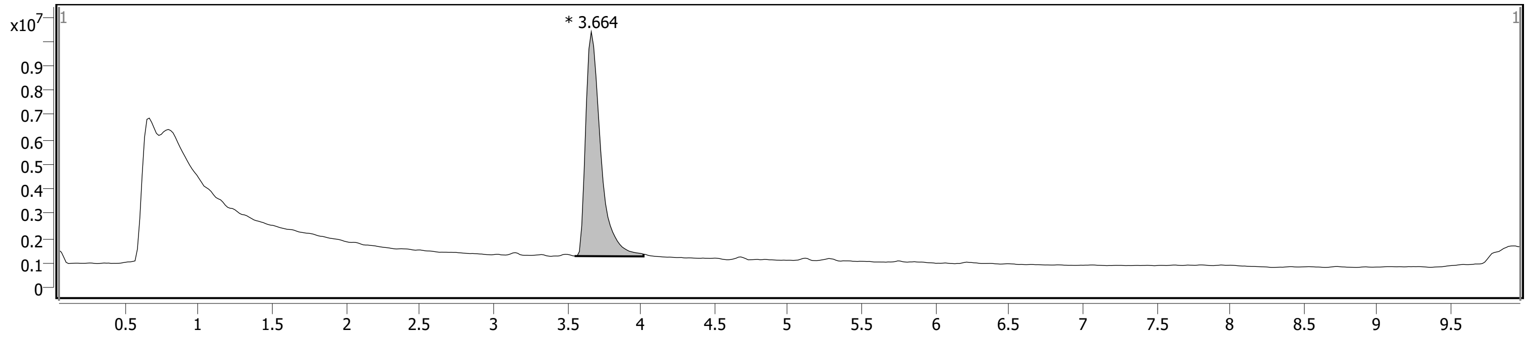

Counts vs. Acquisition Time (min)

Chromatogram Peaks

Peak	Start	RT	End	Height	Area	Area %	SNR
1	3.551	3.664	4.019	9121511	65107273	100.00	

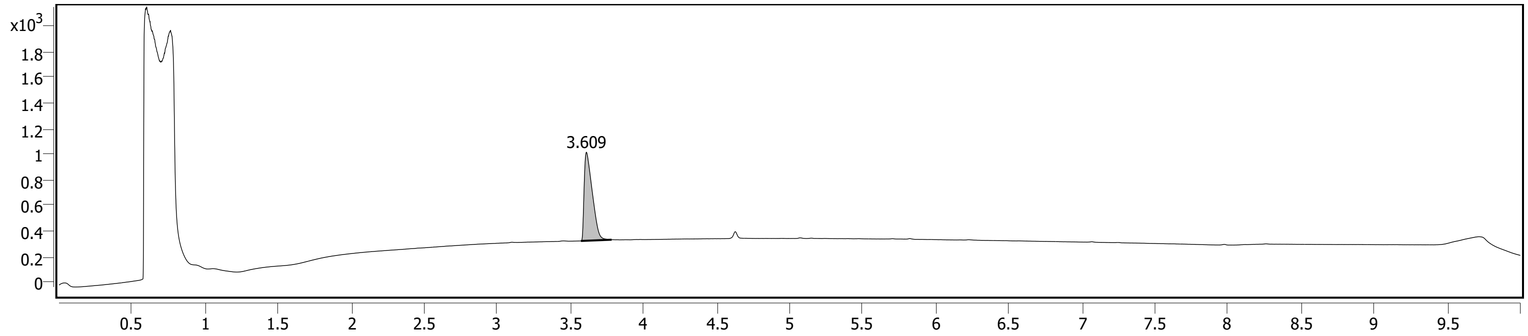

Response Units vs. Acquisition Time (min)

Chromatogram Peaks

Peak	Start	RT	End	Height	Area	Area %	SNR
1	3.572	3.609	3.782	692	2670		

Sample Spectra

+ Scan (rt: 3.600-3.809 min) **Peak 1 from + TIC Scan**

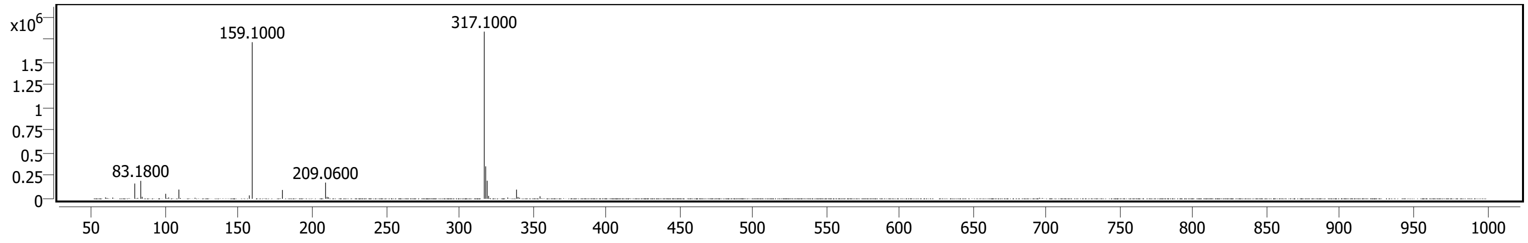

Counts vs. Mass-to-Charge (m/z)

Spectrum Peaks

m/z	Z	Abund	Abund %	m/z (Calc)	Diff (ppm)	Ion Species	Formula	Ion Type
79.1000		168232	9.16					
83.1800		197568	10.75					
84.1500		22254	1.21					
100.1900		55233	3.01					
109.1400		102082	5.56					
157.1000		37706	2.05					
159.1000		1720992	93.68					
179.6400	2	97439	5.30					
209.0600	1	178531	9.72					
210.0700	1	21679	1.18					
317.1000	1	1837019	100.00					
318.1000	1	356869	19.43					
319.1000	1	198914	10.83					
320.1000		32568	1.77					
339.1000	1	101264	5.51					
340.0800	1	20104	1.09					
355.1000		28023	1.53					

MassHunter Qual 10.0
(End of Report)

Analysis Report

Sample Information

**Name**	3	**Structure**	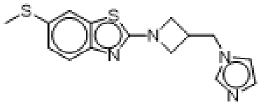
**Sample ID**			
**Instrument**	LC-SQ		
**MS Type**	Q		
**Inj. Vol. (ul)**	-1		
**Position**	P1-A8		
**Plate Pos.**		**MW**	345.34
**Operator**	SYSTEM	**Result Summary**	

Sample Chromatograms

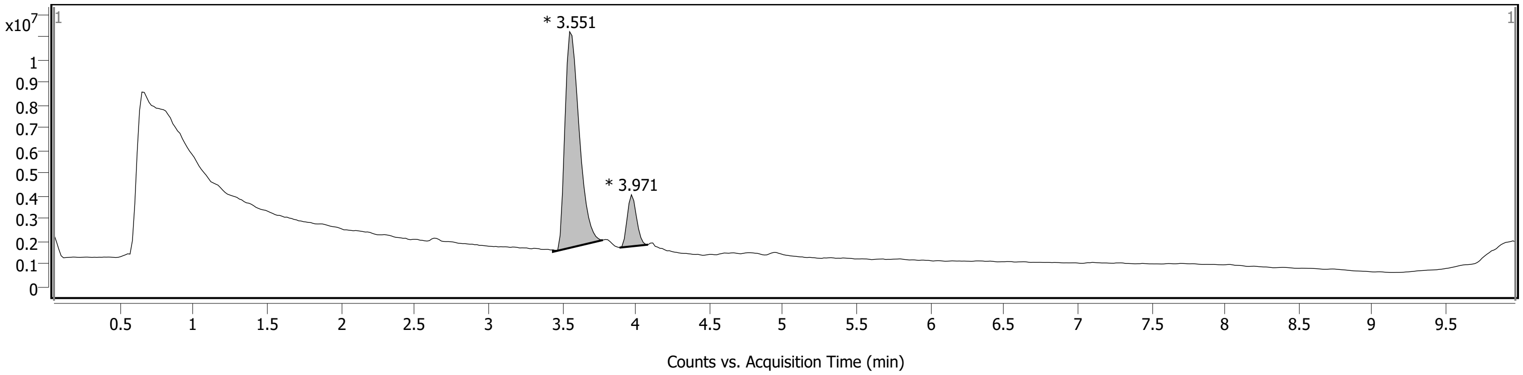

Chromatogram Peaks

Peak	Start	RT	End	Height	Area	Area %	SNR
1	3.438	3.551	3.777	9494708	64263757	100.00	
2	3.890	3.971	4.083	2269556	9915874	15.43	

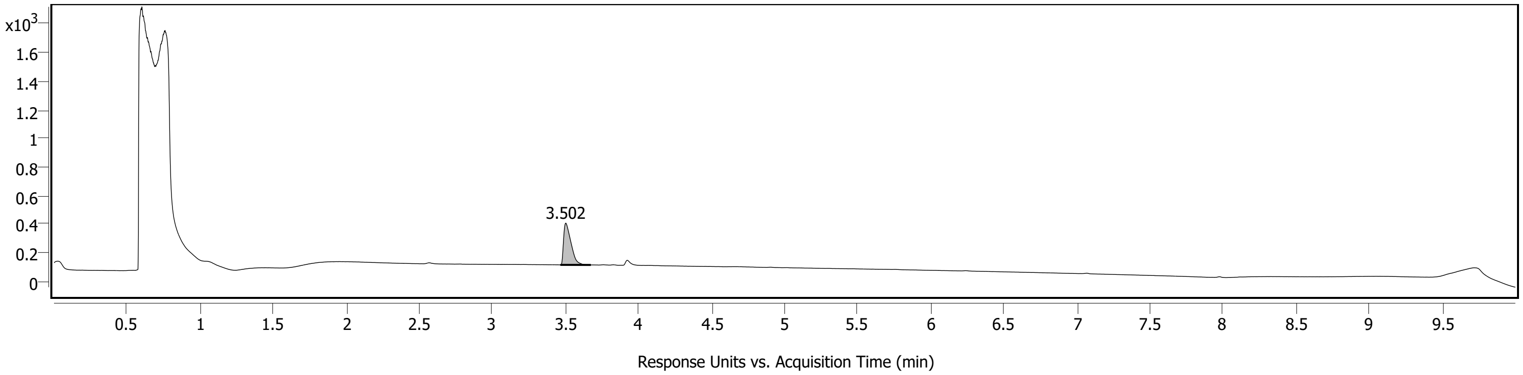

Chromatogram Peaks

Peak	Start	RT	End	Height	Area	Area %	SNR
1	3.465	3.502	3.674	292	1025		

Sample Spectra

+ Scan (rt: 3.503-3.680 min) **Peak 1 from + TIC Scan**

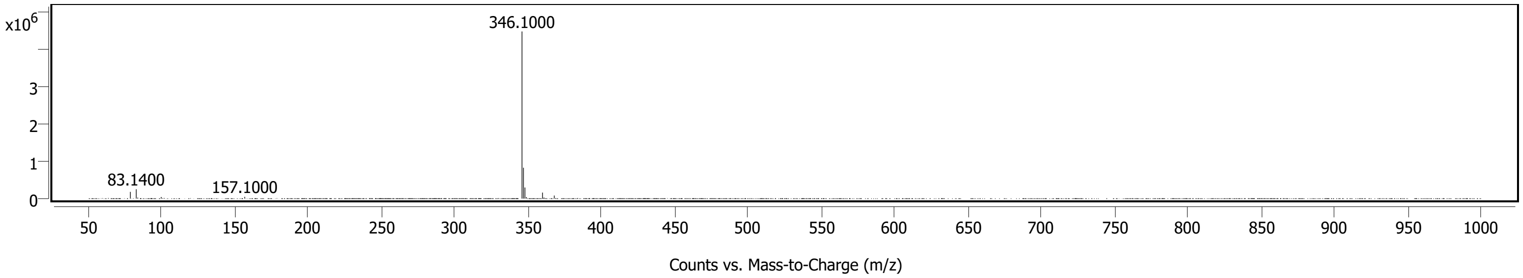

Spectrum Peaks

m/z	Z	Abund	Abund %	m/z (Calc)	Diff (ppm)	Ion Species	Formula	Ion Type
79.1000		183078	4.07					
83.1400		253728	5.64					
100.1800		45426	1.01					
157.1000		49547	1.10					
346.1000	1	4500438	100.00					
347.1000	1	832427	18.50					
348.1000	1	300516	6.68					
360.1000		163480	3.63					
368.1000		83880	1.86					

+ Scan (rt: 3.922-4.035 min) **Peak 2 from + TIC Scan**

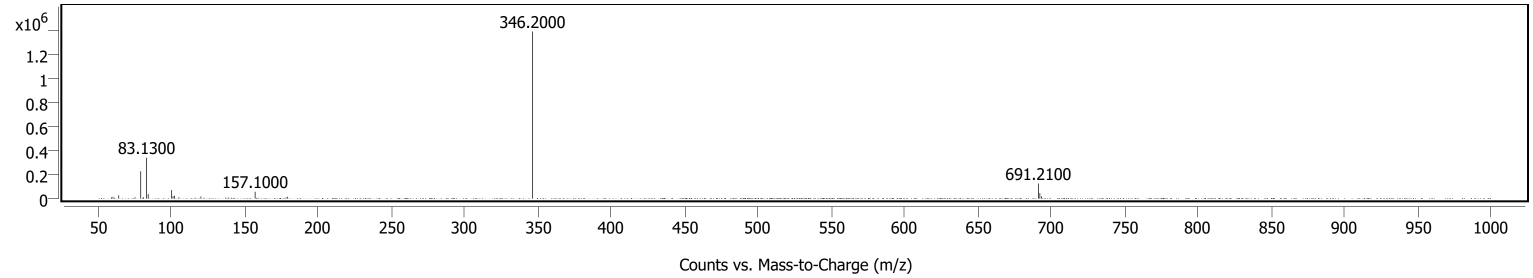

Spectrum Peaks

m/z	Z	Abund	Abund %	m/z (Calc)	Diff (ppm)	Ion Species	Formula	Ion Type
60.1800		15440	1.11					
64.1000		27409	1.97					
79.1000		227192	16.36					
83.1300		339720	24.46					
84.1800		38649	2.78					
100.2000		70986	5.11					
101.1000		20826	1.50					
102.2000		24001	1.73					
120.1100		17545	1.26					
157.1000		57319	4.13					
179.0500		15676	1.13					
346.2000		1389056	100.00					
691.2100	1	125142	9.01					
692.2000	1	46777	3.37					
693.2400	1	19425	1.40					

MassHunter Qual 10.0
(End of Report)

Analysis Report

Sample Information

**Name**	4	**Structure**	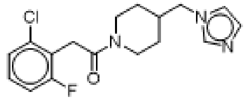
**Sample ID**			
**Instrument**	LC-SQ		
**MS Type**	Q		
**Inj. Vol. (ul)**	-1		
**Position**	P1-B1		
**Plate Pos.**		**MW**	335.81
**Operator**	SYSTEM	**Result Summary**	

Sample Chromatograms

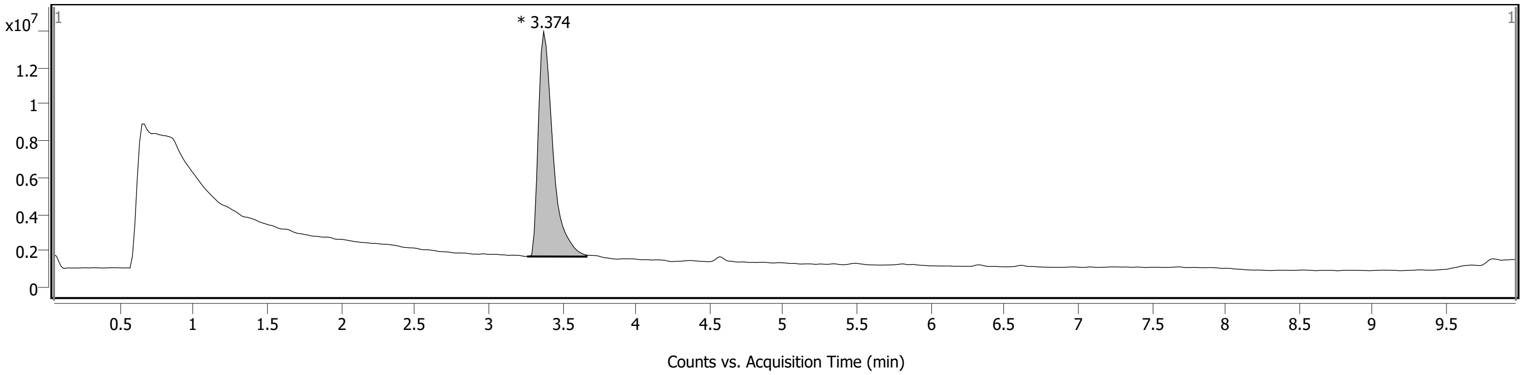

Chromatogram Peaks

Peak	Start	RT	End	Height	Area	Area %	SNR
1	3.261	3.374	3.664	12266350	84695306	100.00	

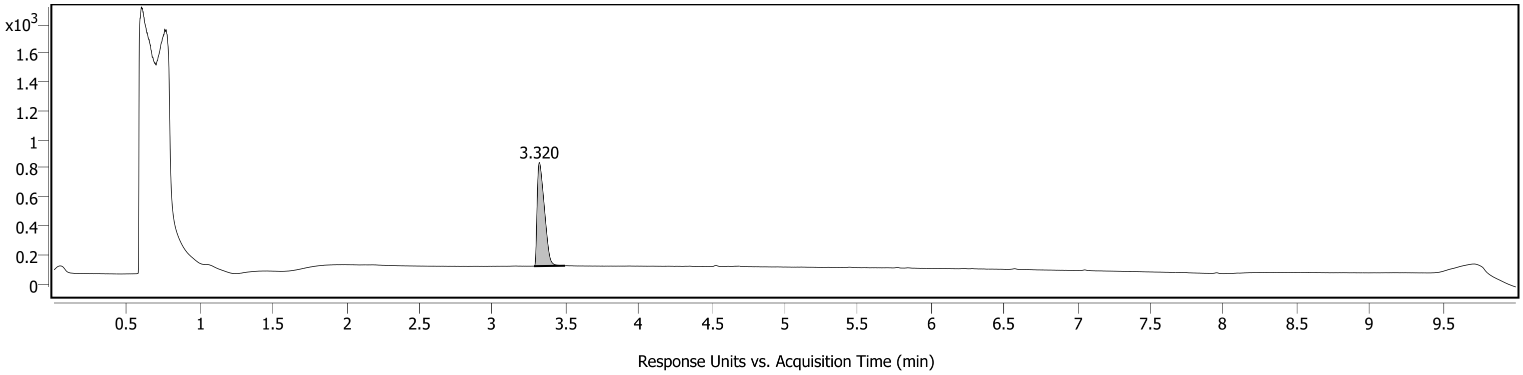

Chromatogram Peaks

Peak	Start	RT	End	Height	Area	Area %	SNR
1	3.284	3.320	3.497	721	2494		

Sample Spectra

+ Scan (rt: 3.309-3.519 min) **Peak 1 from + TIC Scan**

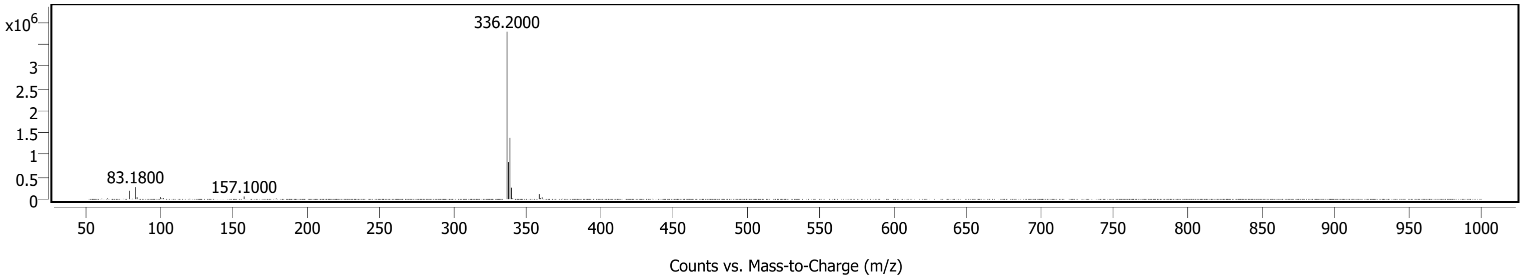

Spectrum Peaks

m/z	Z	Abund	Abund %	m/z (Calc)	Diff (ppm)	Ion Species	Formula	Ion Type
79.1000		191187	5.05					
83.1800		270825	7.16					
84.1900		43462	1.15					
100.1400		50668	1.34					
157.1000		60039	1.59					
336.2000	1	3782382	100.00					
337.2000	1	835849	22.10					
338.2000	1	1388773	36.72					
339.1800	1	260678	6.89					
358.1800		115018	3.04					
360.1500		40548	1.07					

MassHunter Qual 10.0
(End of Report)

Analysis Report

Agilent | Trusted Answers

Sample Information

**Name**	5	**Structure**	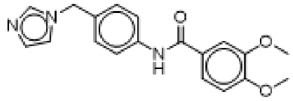
**Sample ID**			
**Instrument**	LC-SQ		
**MS Type**	Q		
**Inj. Vol. (ul)**	-1		
**Position**	P1-B5		
**Plate Pos.**		**MW**	337.38
**Operator**	SYSTEM	**Result Summary**	

Sample Chromatograms

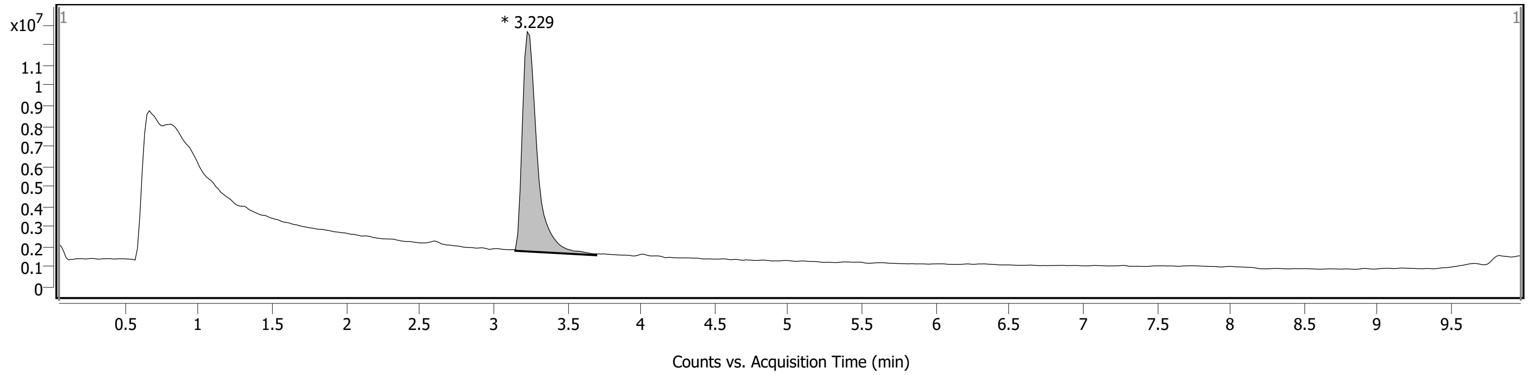

Chromatogram Peaks

Peak	Start	RT	End	Height	Area	Area %	SNR
1	3.148	3.229	3.696	10893542	76508686	100.00	

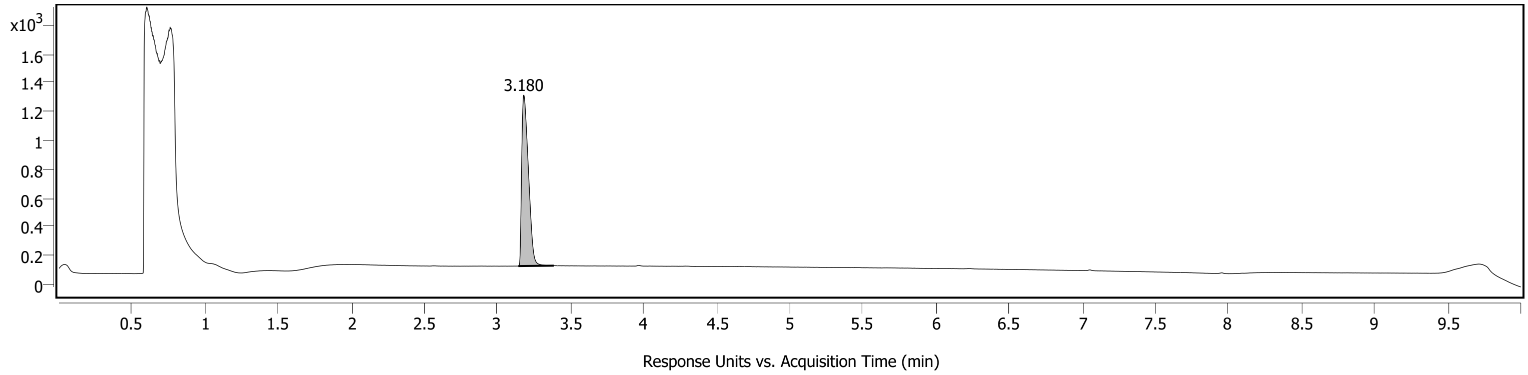

Chromatogram Peaks

Peak	Start	RT	End	Height	Area	Area %	SNR
1	3.143	3.180	3.385	1186	3813		

Sample Spectra

+ Scan (rt: 3.180-3.374 min) **Peak 1 from + TIC Scan**

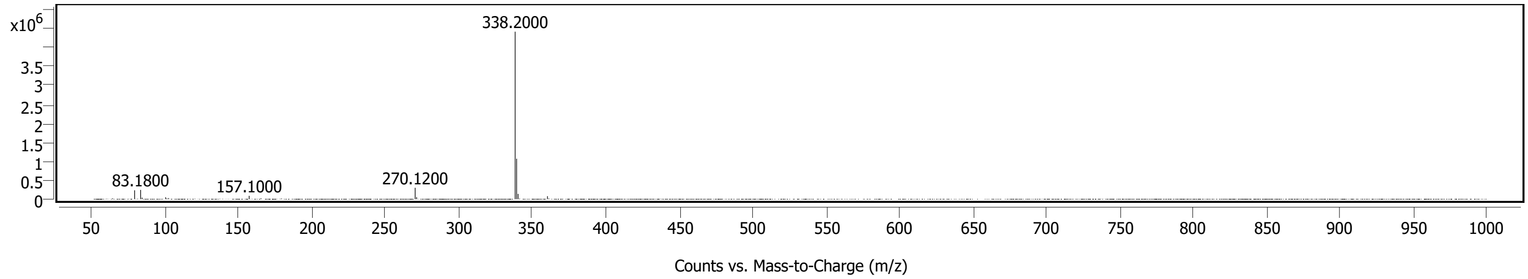

Spectrum Peaks

m/z	Z	Abund	Abund %	m/z (Calc)	Diff (ppm)	Ion Species	Formula	Ion Type
79.1000		234561	5.30					
83.1800		245292	5.55					
100.2000		50391	1.14					
157.1000		89191	2.02					
270.1200	1	301074	6.81					
271.1000	1	53395	1.21					
338.2000	1	4422021	100.00					
339.2000	1	1069243	24.18					
340.2000	1	140673	3.18					
360.2000		78675	1.78					

MassHunter Qual 10.0
(End of Report)

Analysis Report

Sample Information

**Name**	6	**Structure**	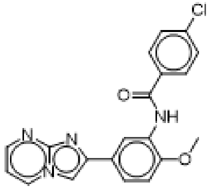
**Sample ID**			
**Instrument**	LC-SQ		
**MS Type**	Q		
**Inj. Vol. (ul)**	-1		
**Position**	P1-B6		
**Plate Pos.**		**MW**	378.82
**Operator**	SYSTEM	**Result Summary**	

Sample Chromatograms

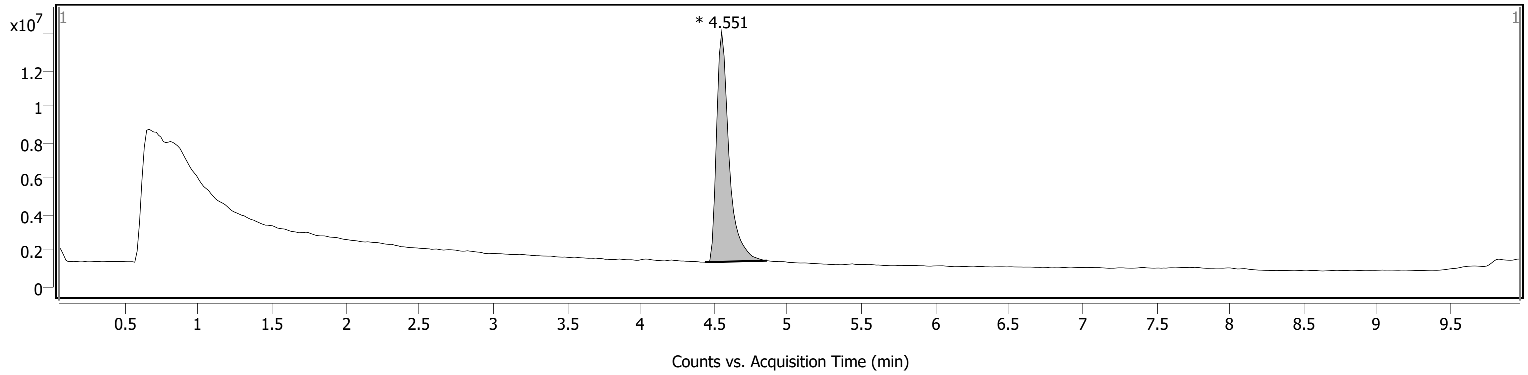

Chromatogram Peaks

Peak	Start	RT	End	Height	Area	Area %	SNR
1	4.438	4.551	4.858	12719909	75029594	100.00	

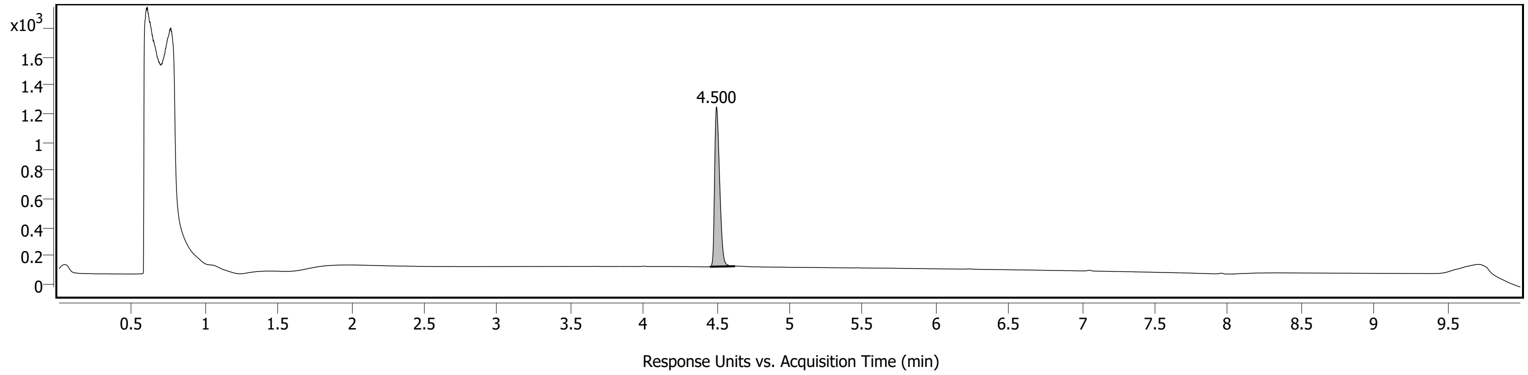

Chromatogram Peaks

Peak	Start	RT	End	Height	Area	Area %	SNR
1	4.461	4.500	4.626	1120	2684		

Sample Spectra

+ Scan (rt: 4.503-4.664 min) **Peak 1 from + TIC Scan**

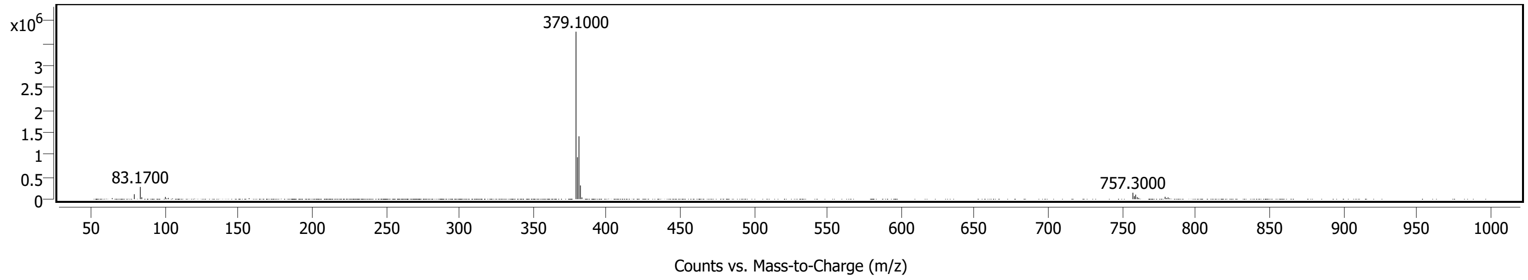

Spectrum Peaks

m/z	Z	Abund	Abund %	m/z (Calc)	Diff (ppm)	Ion Species	Formula	Ion Type
79.1000		111479	2.96					
83.1700		275427	7.32					
84.1900		43695	1.16					
100.1900		50133	1.33					
379.1000	1	3764335	100.00					
380.1000	1	943697	25.07					
381.1000	1	1413050	37.54					
382.1000	1	309158	8.21					
383.1000	1	40063	1.06					
757.3000	1	145412	3.86					
758.2900	1	67627	1.80					
759.2100	1	102236	2.72					
760.2900	1	46447	1.23					
779.2000		54246	1.44					
781.2000		40103	1.07					

MassHunter Qual 10.0
(End of Report)

Analysis Report

Sample Information

**Name**	7	**Structure**	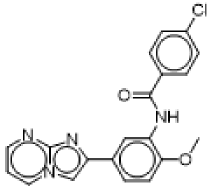
**Sample ID**			
**Instrument**	LC-SQ		
**MS Type**	Q		
**Inj. Vol. (ul)**	-1		
**Position**	P1-B7		
**Plate Pos.**		**MW**	377.38
**Operator**	SYSTEM	**Result Summary**	

Sample Chromatograms

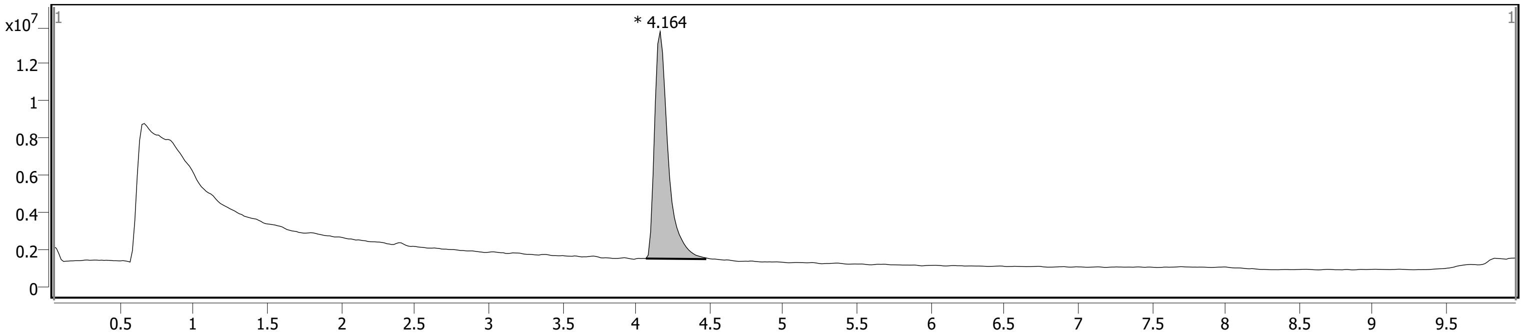

Chromatogram Peaks

Peak	Start	RT	End	Height	Area	Area %	SNR
1	4.067	4.164	4.471	12237516	78946064	100.00	

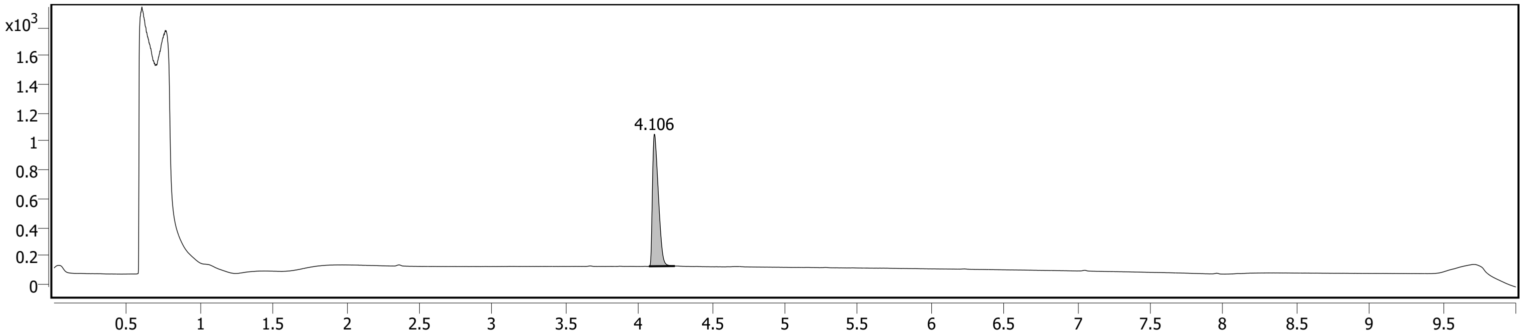

Chromatogram Peaks

Peak	Start	RT	End	Height	Area	Area %	SNR
1	4.070	4.106	4.247	926	2605		

Sample Spectra

+ Scan (rt: 4.100-4.293 min) **Peak 1 from + TIC Scan**

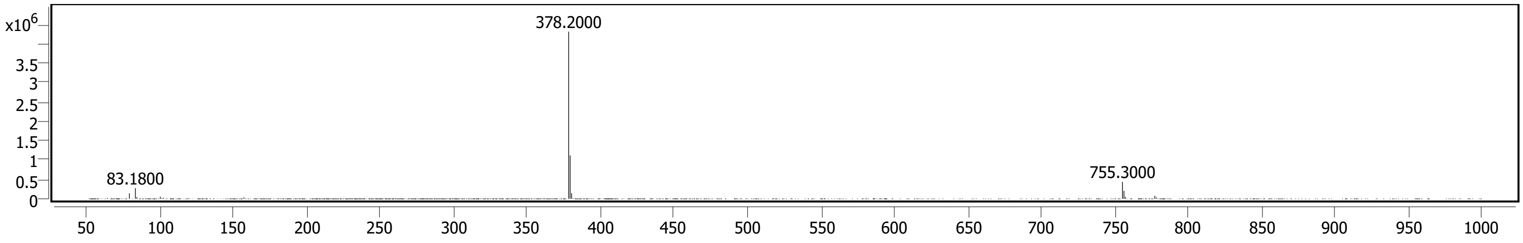

Spectrum Peaks

m/z	Z	Abund	Abund %	m/z (Calc)	Diff (ppm)	Ion Species	Formula	Ion Type
79.1000		142621	3.30					
83.1800		276770	6.41					
84.2000		45432	1.05					
100.1700		52994	1.23					
378.2000	1	4315766	100.00					
379.2000	1	1119690	25.94					
380.2000	1	146208	3.39					
755.3000	1	436932	10.12					
756.3000	1	206231	4.78					
757.3000	1	50451	1.17					
777.3000		76488	1.77					

MassHunter Qual 10.0
(End of Report)

Analysis Report

Sample Information

**Name**	8	**Structure**	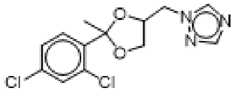
**Sample ID**			
**Instrument**	LC-SQ		
**MS Type**	Q		
**Inj. Vol. (ul)**	-1		
**Position**	P1-B8		
**Plate Pos.**		**MW**	314.17
**Operator**	SYSTEM	**Result Summary**	

Sample Chromatograms

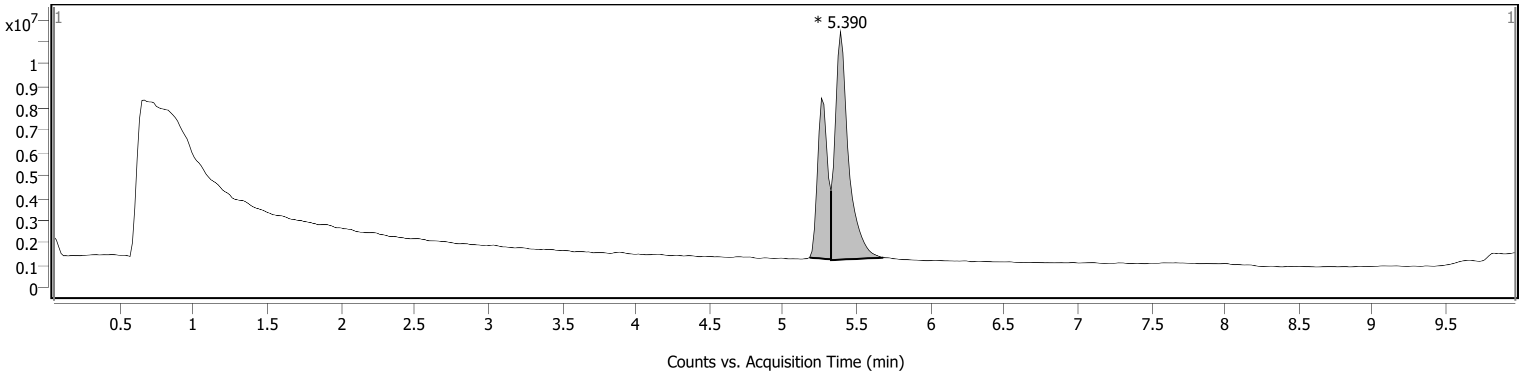

Chromatogram Peaks

Peak	Start	RT	End	Height	Area	Area %	SNR
1	5.180	5.261	5.325	7178916	33962470	51.94	
2	5.325	5.390	5.680	10200856	65389912	100.00	

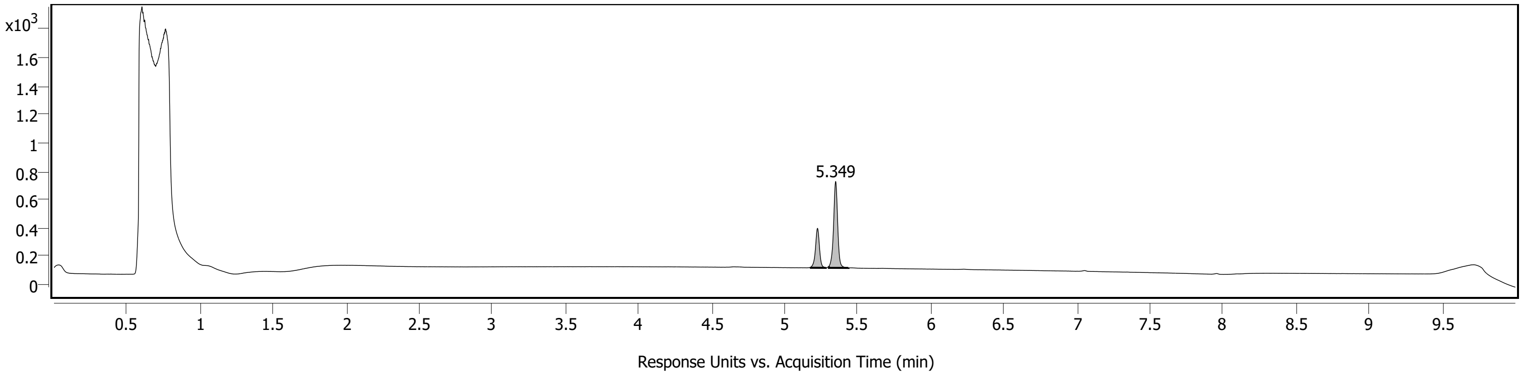

Chromatogram Peaks

Peak	Start	RT	End	Height	Area	Area %	SNR
1	5.179	5.225	5.281	279	534		
2	5.303	5.349	5.437	610	1196		

Sample Spectra

+ Scan (rt: 5.212-5.325 min) **Peak 1 from + TIC Scan**

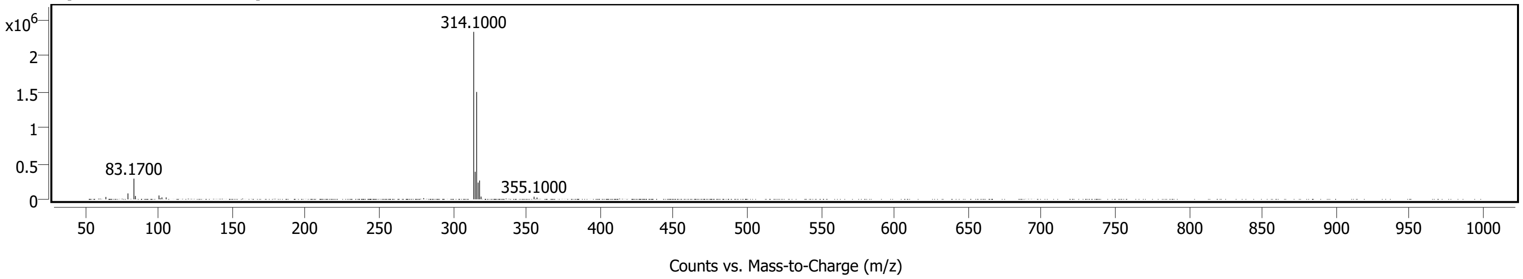

Spectrum Peaks

m/z	Z	Abund	Abund %	m/z (Calc)	Diff (ppm)	Ion Species	Formula	Ion Type
64.1100		33349	1.43					
79.1000		82693	3.54					
83.1700		288976	12.39					
84.1700		47235	2.02					
100.2000		53789	2.31					
102.2000		27278	1.17					
105.1000		27145	1.16					
314.1000	1	2332760	100.00					
315.1000	1	384148	16.47					
316.1000	1	1495664	64.12					
317.1000	1	233346	10.00					
318.1000	1	262294	11.24					
319.0900	1	38814	1.66					
355.1000		36713	1.57					
357.1000		25411	1.09					

+ Scan (rt: 5.325-5.519 min) **Peak 2 from + TIC Scan**

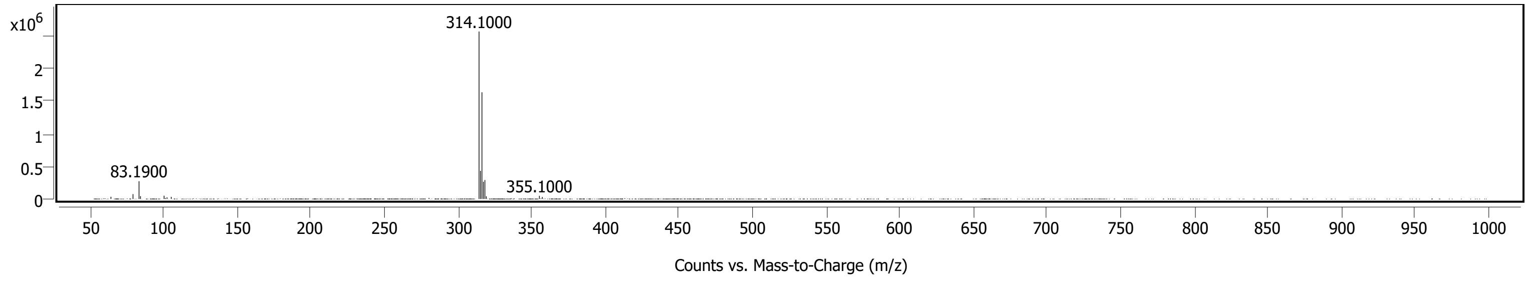

Spectrum Peaks

m/z	Z	Abund	Abund %	m/z (Calc)	Diff (ppm)	Ion Species	Formula	Ion Type
64.1200		34334	1.34					
79.1000		75517	2.94					
83.1900		273300	10.63					
84.1800		44351	1.72					
100.2000		50271	1.95					
105.1000		32662	1.27					
314.1000	1	2571683	100.00					
315.1000	1	432884	16.83					
316.1000	1	1638961	63.73					
317.1000	1	265032	10.31					
318.1000	1	291246	11.33					
319.0900	1	42316	1.65					
355.1000		49503	1.92					
357.1000		31604	1.23					

MassHunter Qual 10.0
(End of Report)

Analysis Report

Sample Information

**Name**	9	**Structure**	
**Sample ID**			
**Instrument**	LC-SQ		
**MS Type**	Q		
**Inj. Vol. (ul)**	-1		
**Position**	P1-B9		
**Plate Pos.**		**MW**	393.83
**Operator**	SYSTEM	**Result Summary**	

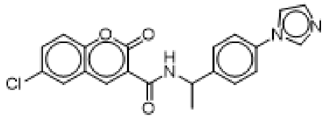

Sample Chromatograms

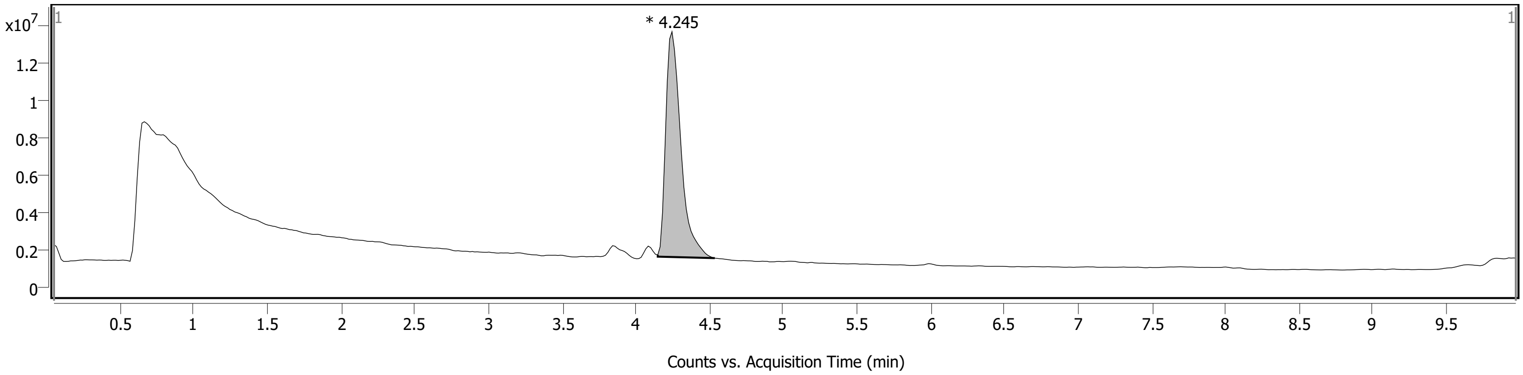

Chromatogram Peaks

Peak	Start	RT	End	Height	Area	Area %	SNR
1	4.148	4.245	4.535	12091299	86533843	100.00	

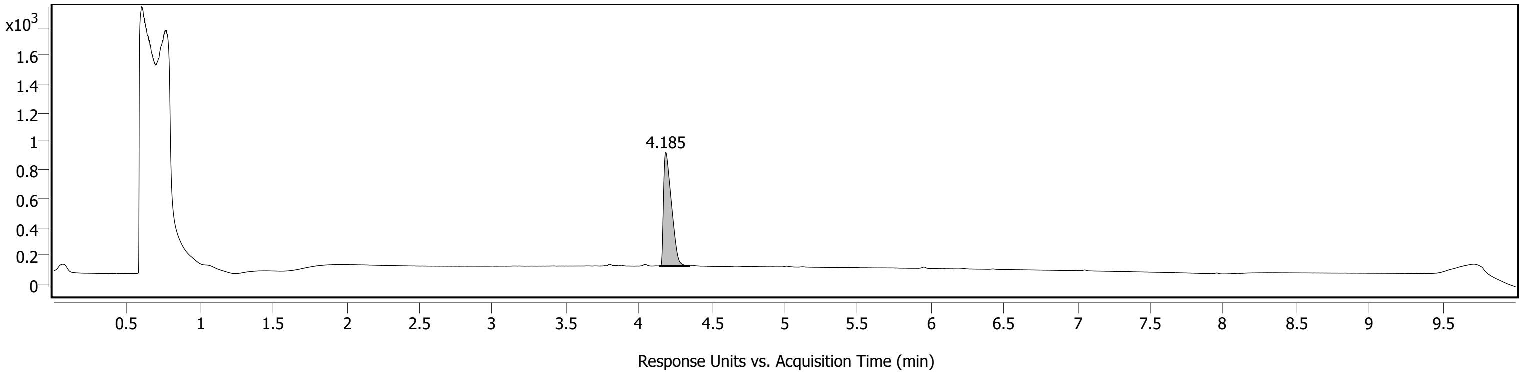

Chromatogram Peaks

Peak	Start	RT	End	Height	Area	Area %	SNR
1	4.140	4.185	4.352	793	2994		

Sample Spectra

+ Scan (rt: 4.180-4.374 min) **Peak 1 from + TIC Scan**

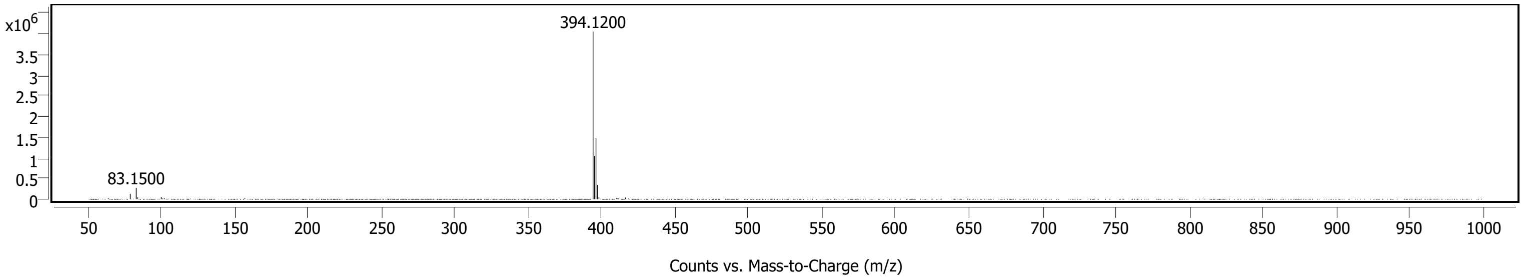

Spectrum Peaks

m/z	Z	Abund	Abund %	m/z (Calc)	Diff (ppm)	Ion Species	Formula	Ion Type
79.1000		129432	3.19					
83.1500		272753	6.73					
84.2000		45969	1.13					
100.1800		49862	1.23					
394.1200	1	4053209	100.00					
395.1000	1	1043200	25.74					
396.1000	1	1477814	36.46					
397.1000	1	347474	8.57					
398.1000	1	48069	1.19					
416.1000		41486	1.02					

MassHunter Qual 10.0
(End of Report)

Analysis Report

Sample Information

**Name**	10	**Structure**	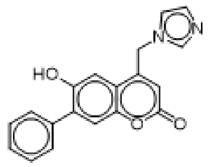
**Sample ID**			
**Instrument**	LC-SQ		
**MS Type**	Q		
**Inj. Vol. (ul)**	-1		
**Position**	P1-C2		
**Plate Pos.**		**MW**	318.33
**Operator**	SYSTEM	**Result Summary**	

Sample Chromatograms

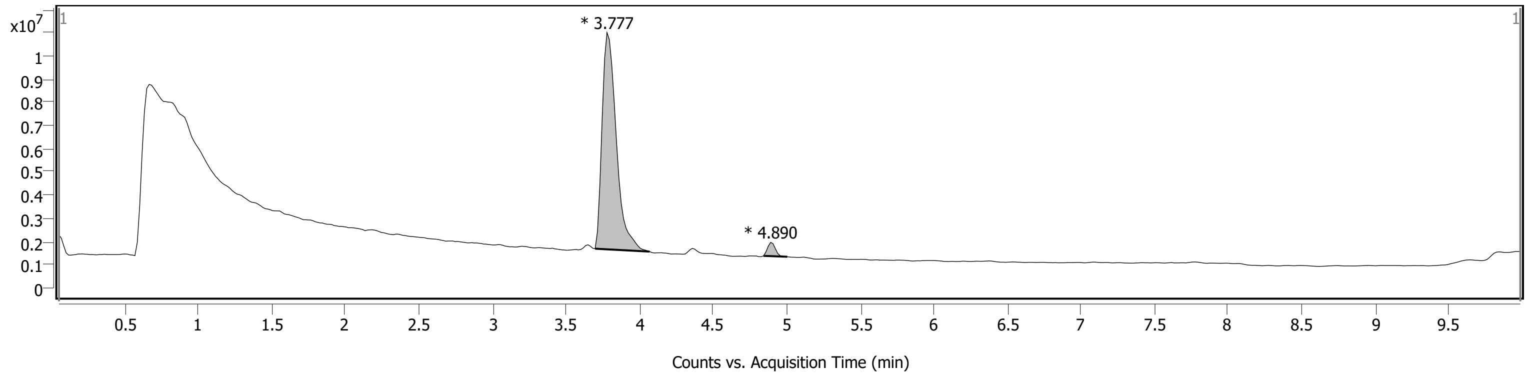

Chromatogram Peaks

Peak	Start	RT	End	Height	Area	Area %	SNR
1	3.696	3.777	4.067	9332618	62948009	100.00	
2	4.841	4.890	5.003	593777	2155844	3.42	

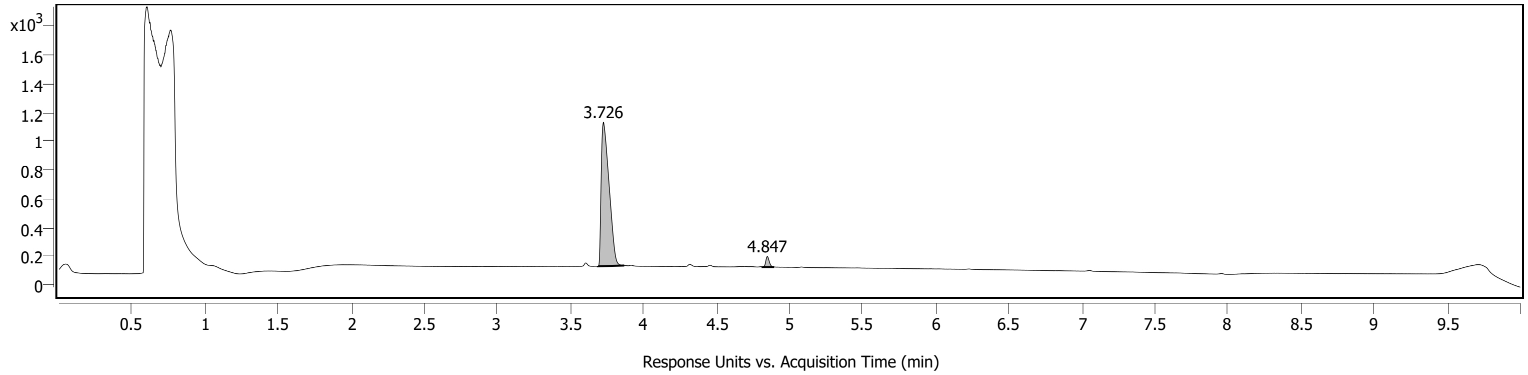

Chromatogram Peaks

Peak	Start	RT	End	Height	Area	Area %	SNR
1	3.688	3.726	3.868	1004	3756		
2	4.815	4.847	4.894	73	122		

Sample Spectra

+ Scan (rt: 3.729-3.906 min) **Peak 1 from + TIC Scan**

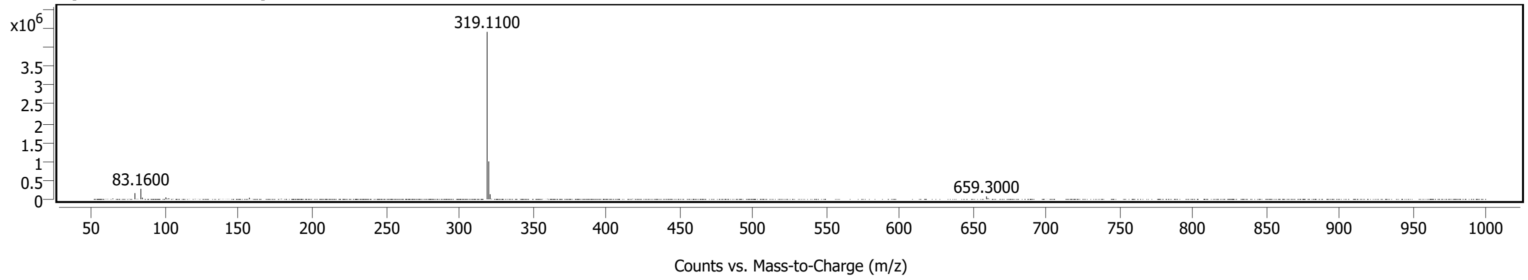

Spectrum Peaks

m/z	Z	Abund	Abund %	m/z (Calc)	Diff (ppm)	Ion Species	Formula	Ion Type
79.1000		163930	3.72					
83.1600		277984	6.30					
84.1900		45291	1.03					
100.1900		52695	1.19					
319.1100	1	4412582	100.00					
320.1900	1	999397	22.65					
321.1500	1	134767	3.05					
659.3000		77570	1.76					

+ Scan (rt: 4.857-4.938 min) **Peak 2 from + TIC Scan**

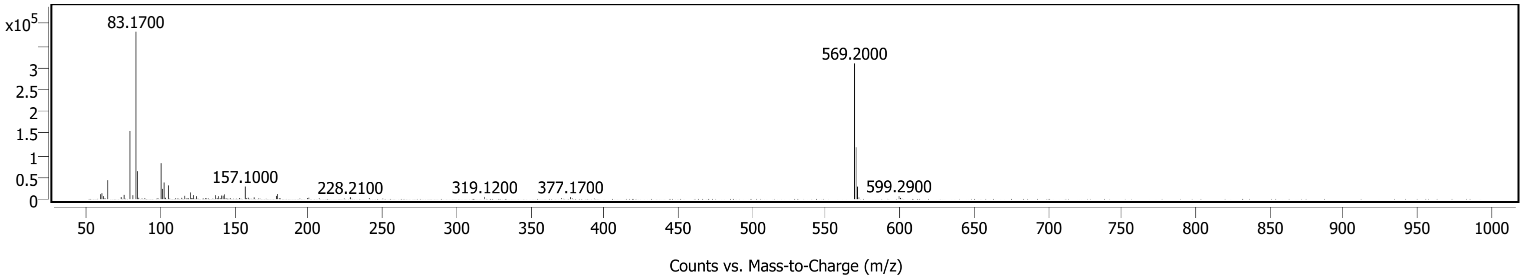

Spectrum Peaks

m/z	Z	Abund	Abund %	m/z (Calc)	Diff (ppm)	Ion Species	Formula	Ion Type
59.2000		11165	2.92					
60.1700		13364	3.50					
61.1700		6896	1.81					
64.1200		43029	11.27					
73.1800		4778	1.25					
75.1800		10075	2.64					
79.1000		155808	40.81					
81.1000		8936	2.34					
83.1700		381760	100.00					
84.2000		63516	16.64					
100.1700		81727	21.41					
101.1000		23760	6.22					
102.2000		37963	9.94					
105.1000		31300	8.20					
116.2000		7836	2.05					
120.1700		15282	4.00					
122.1200		9242	2.42					
124.1200		6625	1.74					
137.1000		8982	2.35					
139.1500		6875	1.80					
141.1200		8295	2.17					
142.1000		8091	2.12					
143.2000		10850	2.84					
157.1000		28781	7.54					
163.1000		3960	1.04					
178.1700		7713	2.02					
179.0000		12111	3.17					
228.2100		4128	1.08					
319.1200		5414	1.42					
377.1700		4529	1.19					
569.2000	1	309568	81.09					
570.2100	1	118139	30.95					
571.2100	1	28650	7.50					
572.2000	1	4062	1.06					
599.2900		7194	1.88					

MassHunter Qual 10.0
(End of Report)

MS Spectra Purity

Data file: 023-P1-C4-S21.D

Acquired by: SYSTEM **Acquired on:** 2024-08-02 16:01:20+02:00

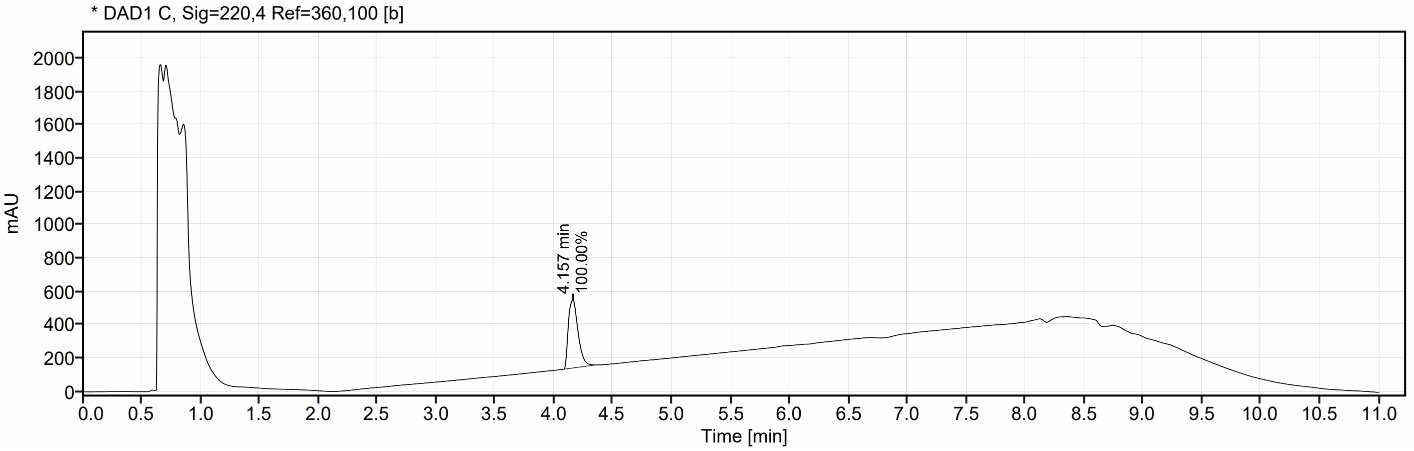

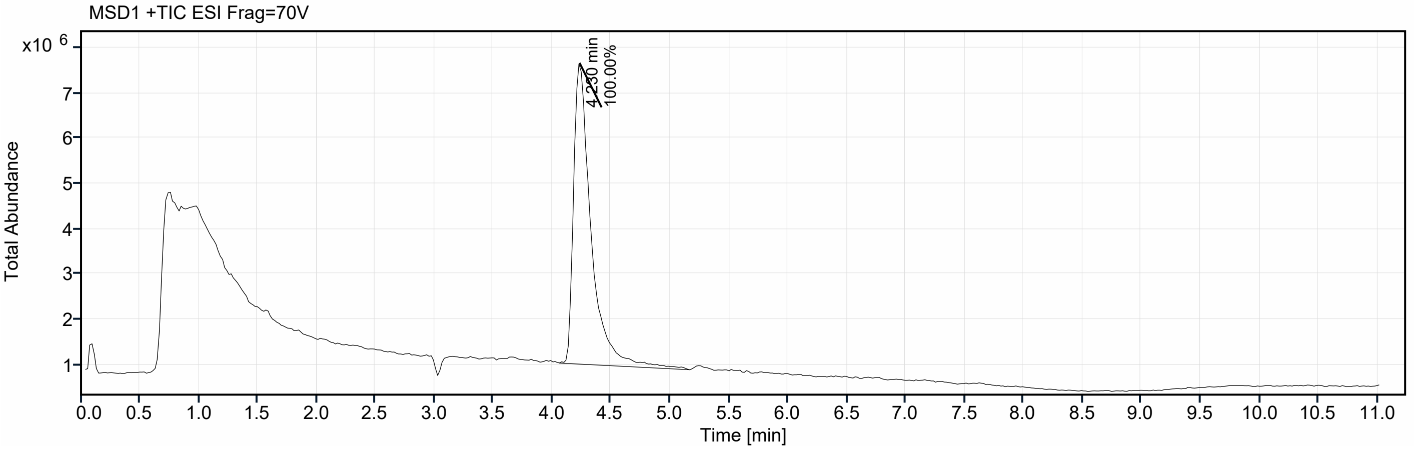

Signal: MSD1 +TIC ESI Frag=70V

RT	m/z	Spectral purity (%)	Peak area	Area %
4.230			68790000	100.00

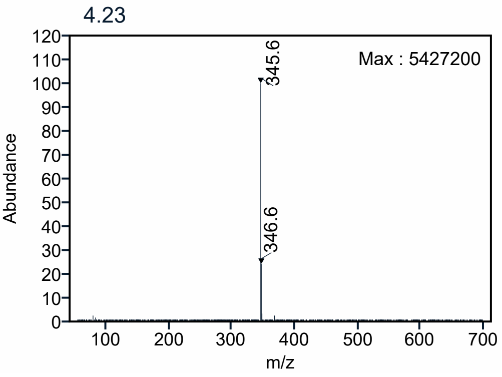

Name 9a

Structure

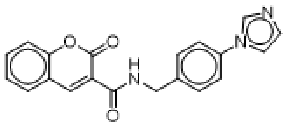

MW 345.36

MS Spectra Purity

Data file: 024-P1-C5-S22.D

Acquired by: SYSTEM **Acquired on:** 2024-08-02 16:15:35+02:00

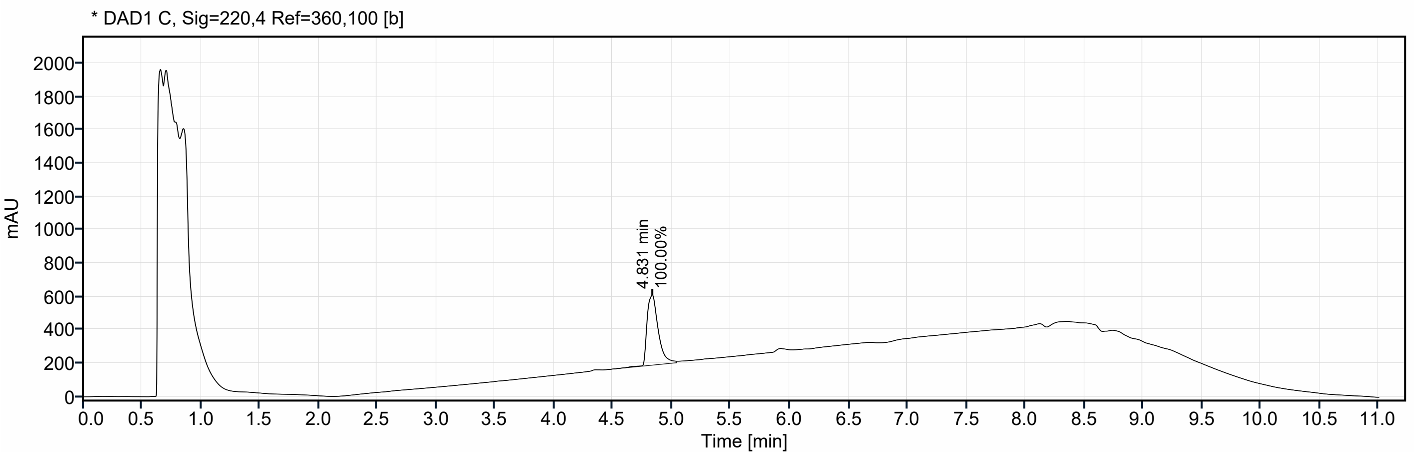

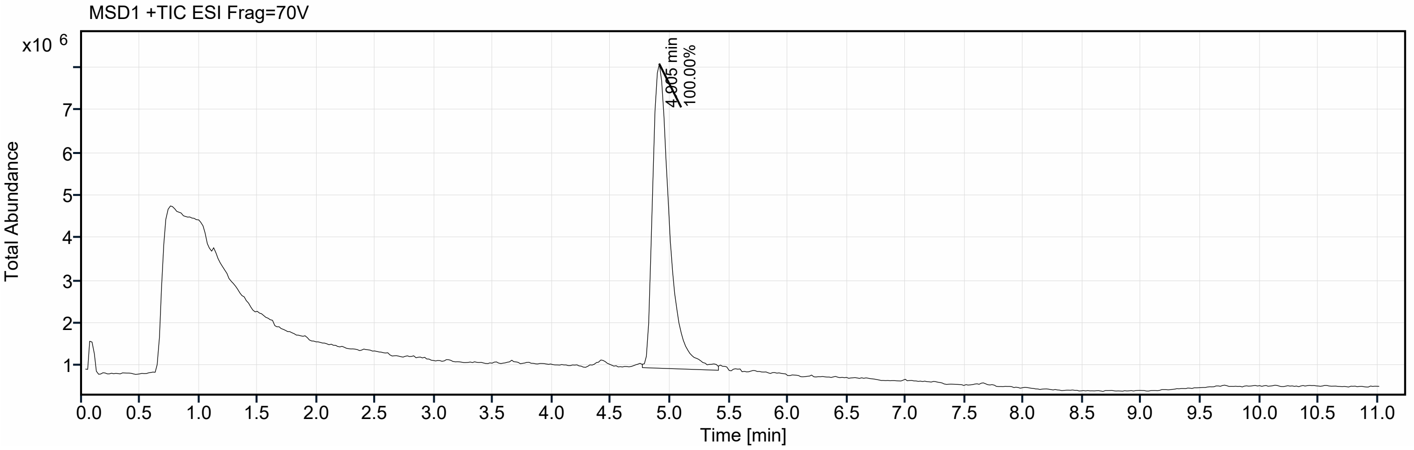

Signal: MSD1 +TIC ESI Frag=70V

RT	m/z	Spectral purity (%)	Peak area	Area %
4.905			71830000	100.00

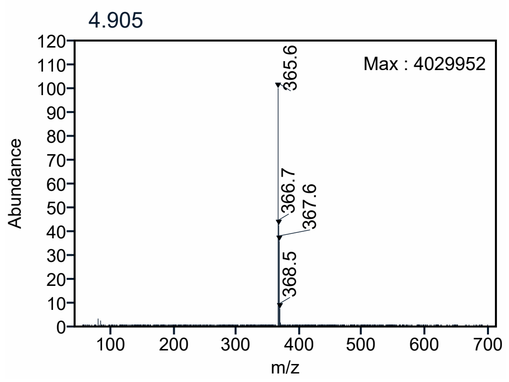

Name 9b

Structure

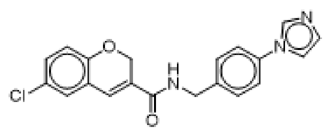

MW 365.82

MS Spectra Purity

Data file: Letrozole__WOLBER_C18_100mm_5_95_neg.D

Acquired by: SYSTEM **Acquired on:** 2023-08-04 14:50:39+02:00

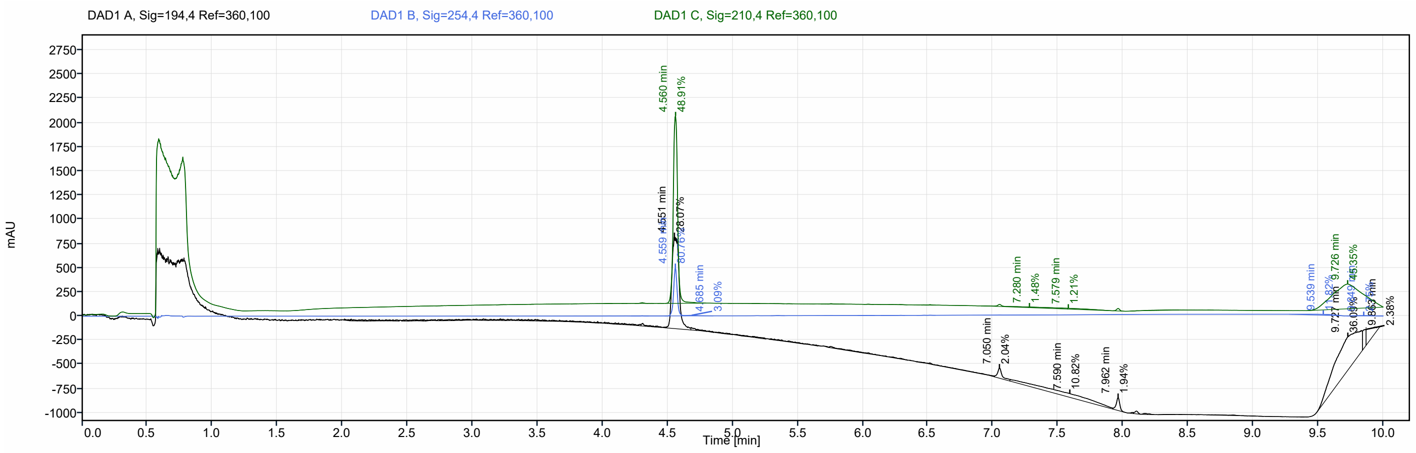

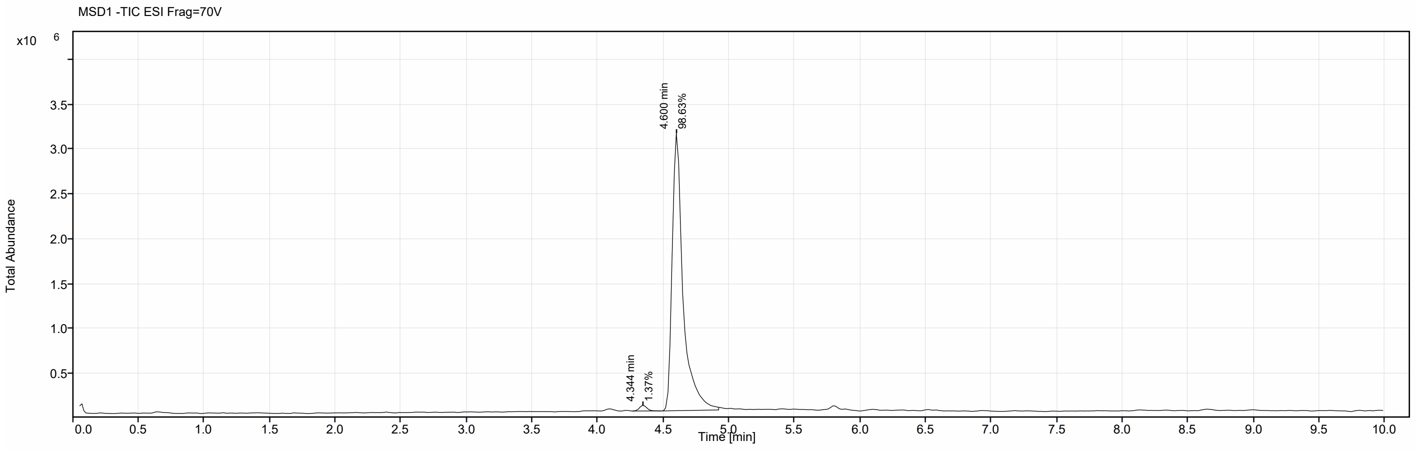

Signal: MSD1 -TIC ESI Frag=70V

RT	m/z	Spectral purity (%)	Peak area	Area %
4.344			249300	1.37

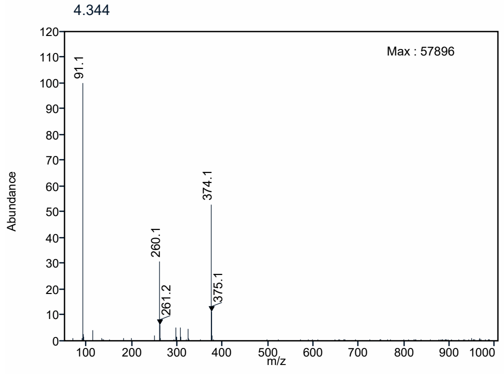

RT	m/z	Spectral purity (%)	Peak area	Area %
4.600			17910000	98.63

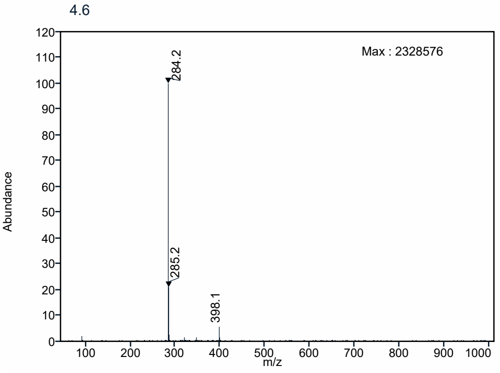

Name Letrozole

Structure

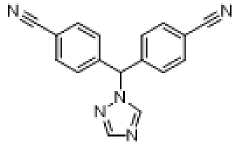

MW 285.31
