# Supplementary material for: Integrated Virtual Screening Approach Identifies New CYP19A1 Inhibitors
Source: J Chem Inf Model. 2025 Mar 19;65(7):3529–43. doi: 10.1021/acs.jcim.5c00204 (PMC12004523; doi:10.1021/acs.jcim.5c00204)
Supplement: Supplementary file 5 — ci5c00204_si_005.pdf [file ci5c00204_si_005.pdf]

Supplementary information

Integrated Virtual Screening Approach Identifies new CYP19A1 Inhibitors

Sijie Liu‡ (1, 2), Jie Wu‡ (3), Ya Chen (2), Clemens Alexander Wolf (1), Matthias Bureik (3), Johannes Kirchmair (2), Mario Andrea Marchisio* (3, 4), Gerhard Wolber* (1)*

1. Pharmaceutical and Medicinal Chemistry (Computer-Aided Drug Design), Institute of Pharmacy, Freie Universität Berlin, 14195 Berlin, Germany
2. Department of Pharmaceutical Sciences, Division of Pharmaceutical Chemistry, Faculty of Life Sciences, University of Vienna, Josef-Holaubek-Platz 2, 1090 Vienna, Austria
3. School of Pharmaceutical Science and Technology, Tianjin University, Tianjin 300072, China
4. Current affiliation: School of Life Science and Health, Northeastern University, 110169 Shenyang, China

Table S1a. Corresponding SMILES and number of active/inactive compounds of the Murcko Scaffolds in the ChEMBL dataset.

Label	SMILES	Scaffold structure	Number of active compounds	Number of inactive compounds
Scaffold 1	c1ccc(CN(c2ccccc2)n2cnnc2)cc1	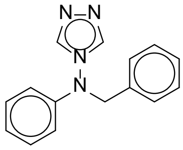	40	0
Scaffold 2	c1ccc(-c2cccc(Cn3cncn3)c2)cc1	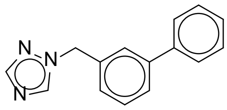	28	0
Scaffold 3	O=S(=O)(NCCc1c(C(c2ccccc2)c2[nH]c3ccccc3c2CCNS(=O)(=O)c2ccccc2)[nH]c2ccccc12)c1ccccc1	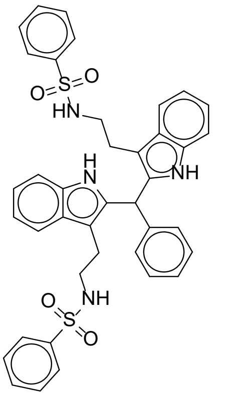	2	24
Scaffold 4	C(=C(c1ccccc1)c1ccccc1)c1ccccc1	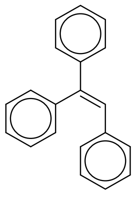	13	11
Scaffold 5	O=C1CCC2C1CCC1C3CCCC=C3CCC21	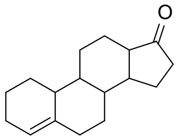	14	7
Scaffold 6	O=C1CC(c2ccccc2)Oc2ccccc21	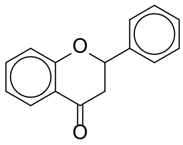	4	17
Scaffold 7	O=S(=O)(c1ccccc1)N1CCCC(Cn2ccnc2)C1	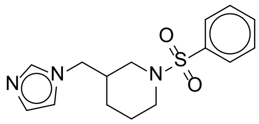	20	0

Scaffold 8	O=c1cc(Cn2ccnc2)c2ccc(OCc3ccccc3)cc2o1	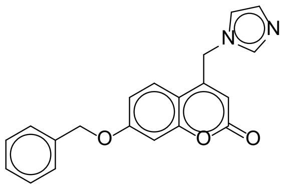	19	0
Scaffold 9	O=C1CCC2C(=CCC3C4CCC(=O)C4CCC23)C1	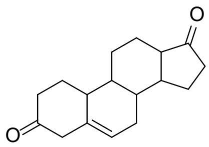	13	6
Scaffold 10	c1ccc(-n2c(Cn3ccnc3)cc3ccccc32)cc1	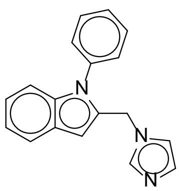	19	0
Scaffold 11	c1ccc(COc2ccccc2)cc1	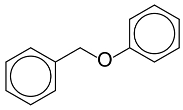	8	11
Scaffold 12	c1ccc(C(c2ccccc2)n2cncn2)cc1	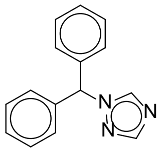	15	2
Scaffold 13	O=C1C=CC2C(=CCC3C2CCC2C(=O)CCC23)C1	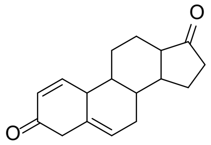	13	3
Scaffold 14	O=c1c2ccccc2oc2cccc(Cn3ccnc3)c12	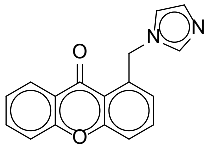	9	5
Scaffold 15	c1ccc(CN(c2cccc(-c3ccccc3)c2)n2cnnc2)cc1	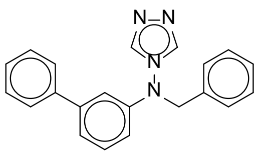	14	0
Scaffold 16	O=S(=O)(NCc1cccc(CNS(=O)(=O)c2ccccc2)c1)c1ccccc1	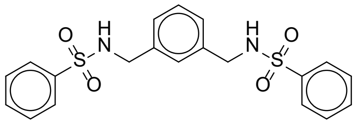	7	6

Scaffold 17	c1ccc(OCc2cn(C(c3ccccc3)c3ccccc3)nn2)cc1	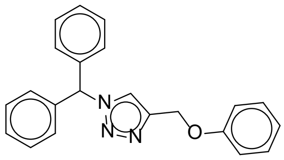	0	12
Scaffold 18	c1ccc(CN(Cc2ccc(-c3ccccc3)cc2)n2ccnc2)cc1	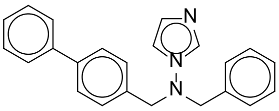	4	8
Scaffold 19	C(=Cc1ccccc1)c1ccccc1	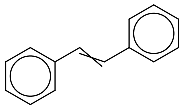	4	8
Scaffold 20	c1ccc(C(c2cc3ccccc3o2)n2cncn2)cc1	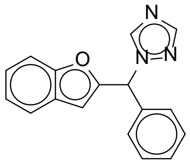	10	2
Scaffold 21	c1ccc(-c2csc(-c3ncccn3)n2)cc1	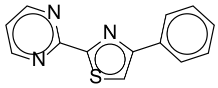	2	10
Scaffold 22	c1ccc(Cn2cc(COc3ccccc3)nn2)cc1	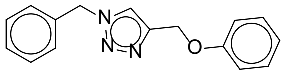	0	12
Scaffold 23	c1ccc(CC(Cc2ccc(-c3ccccc3)cc2)n2ccnc2)cc1	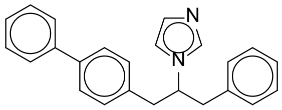	5	6
Scaffold 24	O=C1CCc2cc(C(c3ccccc3)c3ccncc3)cc3c2N1CC3	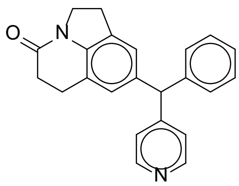	11	0
Scaffold 25	O=C1CCC2C1CCC1C3CCCCC3=CCC21	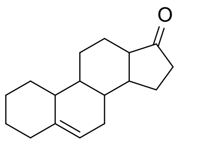	3	8
Scaffold 26	O=c1cc(Cn2ccnc2)c2ccc(Oc3ccccc3)cc2o1	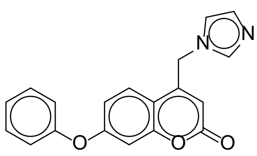	11	0

Table S1b. Corresponding SMILES and number of active/inactive compounds of the Murcko Scaffolds in the PubChem dataset.

Label	SMILES	Scaffold structure	Number of active compounds	Number of inactive compounds
Scaffold A	c1ccccc1	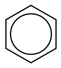	15	222
Scaffold B	Not defined*	Not defined	6	187
Scaffold C	c1ccc(Cc2ccccc2)cc1	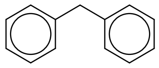	2	32
Scaffold D	c1ccc(Oc2ccccc2)cc1	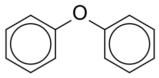	4	19
Scaffold E	c1ccc2ccccc2c1	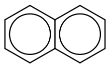	1	14
Scaffold F	c1ccc(-c2ccccc2)cc1	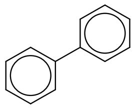	2	12
Scaffold G	c1ccncc1	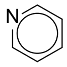	2	11
Scaffold H	O=C(c1ccccc1)c1ccccc1	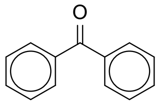	0	12
Scaffold I	c1ccc2c(c1)CCC1C2CCC2CCCC21	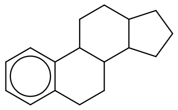	1	10
Scaffold J	c1ccc(Nc2ccccc2)cc1	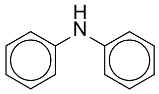	0	10
Scaffold K	c1ccc2ncccc2c1	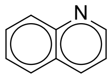	1	9
Scaffold L	O=C(Cc1ccccc1)NC1C(=O)N2CCSC12	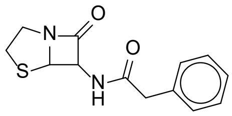	0	9
Scaffold M	O=C1CCC2C(=CCC3C4CCCC4CCC23)C1	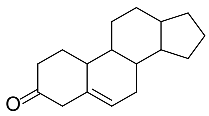	0	9
Scaffold N	O=C(Nc1ccccc1)c1ccccc1	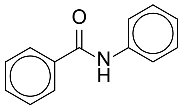	0	9

Scaffold O	C1CCC2C(C1)CCC1C3CCCC3CCC21	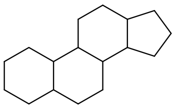	0	8
Scaffold P	O=S1(=O)NCNc2ccccc21	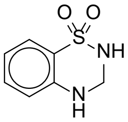	0	8
Scaffold Q	C1CCC(OC2CCCC(OC3CCCCO3)C2)OC1	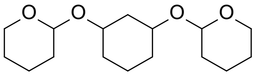	0	8
Scaffold R	O=P(Oc1ccccc1)(Oc1ccccc1)Oc1ccccc1	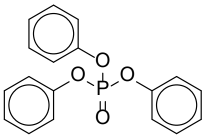	1	6
Scaffold S	c1ccc2c(c1)Nc1ccccc1S2	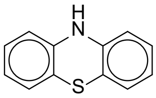	0	7
Scaffold T	O=C(OCc1cccc(Oc2ccccc2)c1)C1CC1	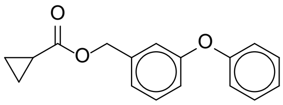	1	6
Scaffold U	c1ccc2[nH]ccc2c1	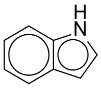	0	7
Scaffold V	c1ncc2ncn(C3CCCO3)c2n1	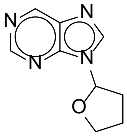	1	6
Scaffold W	C1=CC2C3Cc4cccc5c4C2(CCN3)C(C1)O5	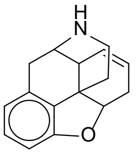	0	7

*Scaffold B is a collection of molecules that could not be defined by Murkuo scaffold, for example: 'CCCCCCCC[P+](CCCCCCCC)(CCCCCCCC)CCCCCCCC', 'CC(C)N(C(=O)SCC(Cl)=CCl)C(C)C', 'CCCCCCCC[N+](CCCCCCCC)(CCCCCCCC)CCCCCCCC'

Table S2 . Relative potency of candidate inhibitors in comparison to letrozole.

	**8**	**9**	**9a**	**9b**	Letrozole	Control
First experiment (A. U.)	10279.26	1950.52	8591.98	9926.37	3161.25	18486.46
Second experiment (A. U.)	10872.42	2721.71	8655.96	9960.87	2909.28	16379.66
Third experiment (A. U.)	8245.57	2143.51	8370.78	9741.93	2581.68	16658.96
Mean	9799.08	2271.91	8539.57	9876.39	2884.07	17175.03
SD	1124.87	327.67	122.18	96.12	237.28	934.31
Relative potency (%)	57±7	13±2	50±1	58±1	17±1	100±5

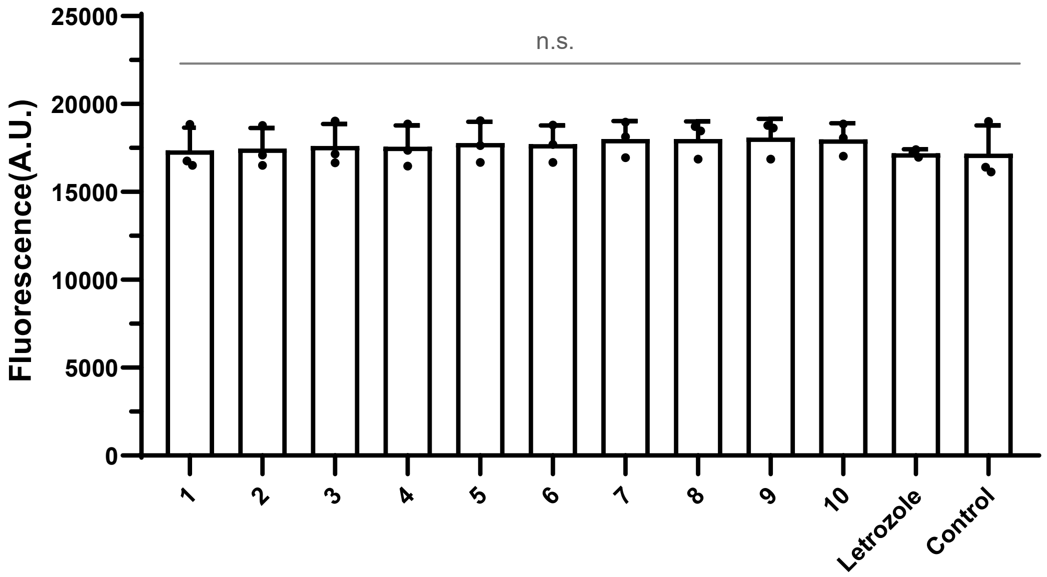

Figure S1. Control experiment: 10 μM samples of inhibitors **1** to **10** were incubated with the biosensor yeast strain in the presence of 30 nM testosterone and 30 nM β-estradiol. No effect was observed as expected.

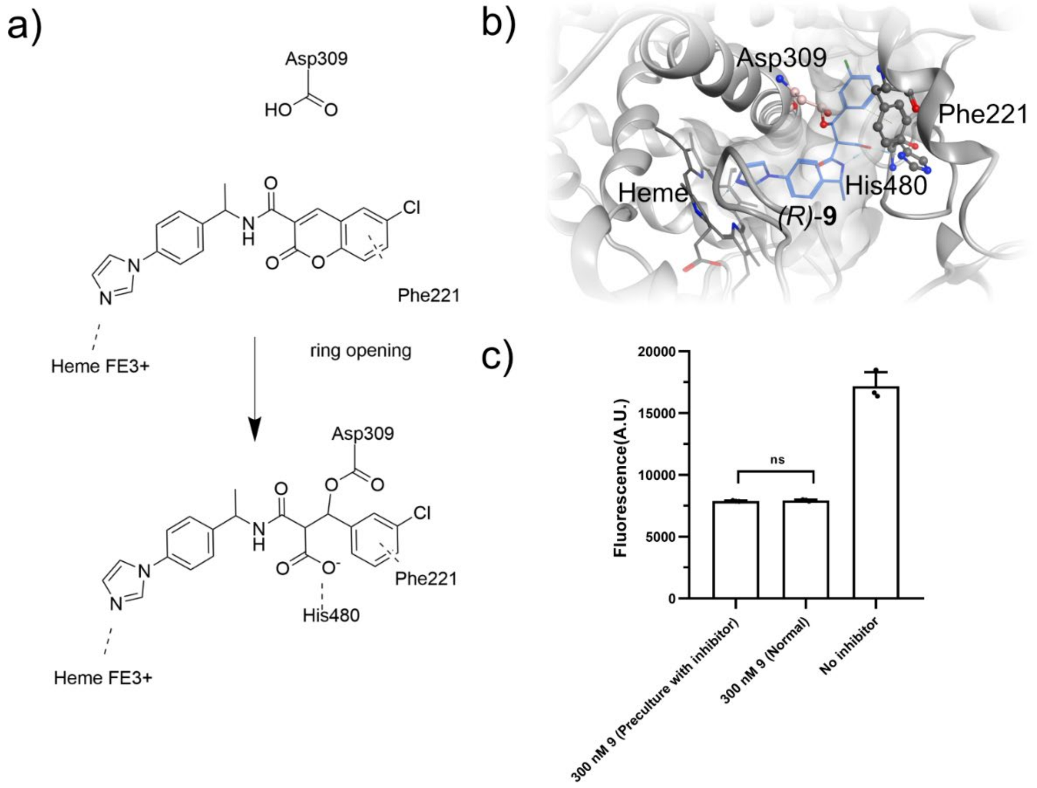

Figure S2. (a) Schematic illustration of the hypothesis that compound **9** covalently binds to human CYP19A1, (b) 3D model of the hypothesis of the covalent binding mode after ring opening reaction and the covalent binding. (c) Inhibition with and without inhibitor pre-incubation culture with compound **9.**

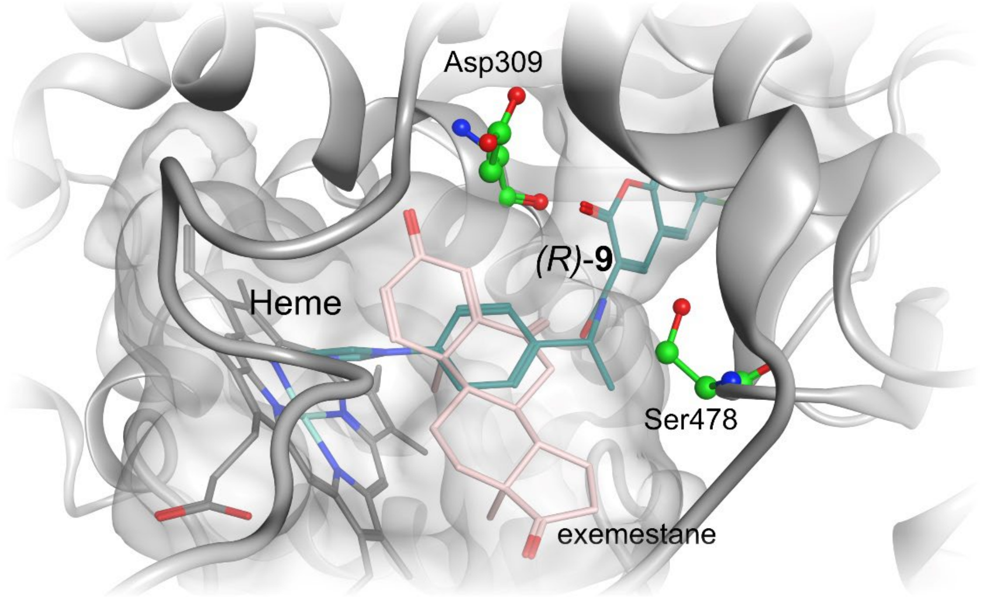

Figure S3. Comparison of *(R)*-**9** (turquoise) and exemestane(pink) in the active site of CYP19A1.
